# Enhancing cancer classification accuracy with a self-attention network using panel capture sequencing data

**DOI:** 10.1093/bib/bbag120

**Published:** 2026-03-23

**Authors:** Yi Jia, Chan Zhang, Han Zhang, Kang Dong, Yuruo Hu, Yinan Wang, Zicheng Zhao

**Affiliations:** Key Laboratory for Early Diagnosis and Biotherapy of Malignant Tumors in Children and Women, Dalian Women and Children’s Medical Group, 154 Zhong Shan Road, Xigang, Dalian 116012, China; Shenzhen Byoryn Technology Co., Ltd, No. 32, Shihua Road, Futian District, Shenzhen 518000, China; Key Laboratory for Early Diagnosis and Biotherapy of Malignant Tumors in Children and Women, Dalian Women and Children’s Medical Group, 154 Zhong Shan Road, Xigang, Dalian 116012, China; Department of Forensic Medicine, Guizhou Medical University, No. 9, Beijing Road, Yunyan District, Guiyang 550025, China; Shenzhen Byoryn Technology Co., Ltd, No. 32, Shihua Road, Futian District, Shenzhen 518000, China; Shenzhen Byoryn Technology Co., Ltd, No. 32, Shihua Road, Futian District, Shenzhen 518000, China; OmicLab Ltd., Unit 917, 9/F, Building 19W, No. 19 Science Park West Avenue, Hong Kong Science Park, Pak Shek Kok, N.T., HongKong, China; Shenzhen Byoryn Technology Co., Ltd, No. 32, Shihua Road, Futian District, Shenzhen 518000, China

**Keywords:** cancer classification, self-attention Conv1D network, single nucleotide mutation

## Abstract

Cancer classification is pivotal for precision oncology, yet traditional methods struggle with the molecular heterogeneity of tumors. Our study introduces a self-attention based Conv1D machine learning network designed for panel capture sequencing data, which is more commonly used in clinical settings. Combining clinical capture sequencing data and The Cancer Genome Atlas data, we achieved an overall classification accuracy of over 90%, with precision rates reaching 100% for cervical and gastric cancers. Additionally, recall rates were highest at 95.79% for gastric cancer and lowest at 77.46% for cervical cancer, demonstrating robust performance across various cancer types. The model identified key genes such as *C3orf36*, *JHY*, and *TASP1*, showing significant differences in mutation counts across cancers. High-impact gene enrichment analysis highlighted critical pathways like acute myeloid leukemia and adipocytokine signaling. This approach not only significantly improves the precision of cancer classification, demonstrating the potential for clinical application, but also enhances our understanding of cancer biology.

## Introduction

Cancer is a complex disease characterized by uncontrolled cell growth, often resulting in severe morbidity and mortality if not detected early. Accurate cancer classification is essential for precision oncology, as it guides targeted therapeutic strategies [1, 2]. Traditional methods, such as histopathology, have limitations in capturing the molecular heterogeneity of tumors, necessitating more advanced approaches that leverage genetic information for precise categorization and personalized treatment strategies.

The complexity of genomic data poses significant challenges in distinguishing between pathogenic variants and benign polymorphisms, and silent mutations can further complicate predictive tasks in cancer diagnosis and treatment planning [3–5]. The inherent heterogeneity within cancer genomes means that even patients with the same cancer subtype may exhibit significant variability in mutational landscapes, complicating clinical decision-making [6, 7]. Next-generation sequencing (NGS) has proven invaluable in identifying novel and rare cancer mutations across various cancer types, such as lung, colorectal, and breast cancers, leading to the development of targeted treatments with improved outcomes [8–11]. Additionally, NGS has facilitated molecular profiling of solid tumors, demonstrating high diagnostic yields in cancers like pancreatic and endometrial cancer [12].

Current cancer classification systems, predominantly based on anatomical origin and histology, often fail to capture the full molecular heterogeneity of tumors, resulting in suboptimal treatment strategies and variable patient outcomes. For instance, breast cancers with similar TNM characteristics can exhibit divergent molecular profiles, leading to different responses to therapy [13–15]. This disconnect underscores the need for sophisticated approaches that accurately reflect the biological diversity of tumors. Traditional methods often group heterogeneous cancers together, obscuring crucial molecular differences and limiting the effectiveness of targeted therapies [16].

The integration of advanced analytical techniques, such as artificial intelligence (AI) and machine learning (ML), is crucial for addressing these limitations [17, 18]. AI and ML can efficiently process large datasets and identify complex patterns that are not readily apparent through traditional methods [19]. AI has been pivotal in deciphering complex genetic mutations and signaling pathways in cancer, enhancing our understanding of its molecular intricacies [20, 21]. For example, AI has been used to classify lung cancer types with high accuracy, demonstrating its potential to improve cancer diagnosis and treatment planning [22]. Studies have shown that AI-driven models can identify novel cancer subtypes and predict patient responses to treatment more effectively than traditional methods [23]. Additionally, AI has been applied in predicting adverse events and survival outcomes in patients receiving immunotherapy, further supporting its role in clinical decision-making. For example, DeepCues utilizes convolutional neural networks (CNNs) to classify seven different types of major cancers using raw whole-exome sequencing data from The Cancer Genome Atlas (TCGA), achieving an overall accuracy of 77.6% [24]. While DeepCues represents a significant advancement, its reliance on raw sequencing data limits its applicability in real clinical settings where panel capture sequencing data is more commonly used. This highlights the need for alternative approaches that can better capture the nuanced patterns within genomic data and provide more flexibility for clinical use.

Our study introduces a groundbreaking self-attention based Conv1D machine learning network specifically designed for panel capture sequencing data, achieving an impressive overall classification accuracy of 90.20%. This model demonstrated high precision and recall rates, with colon cancer classification reaching 89.61% precision and 93.24% recall, and lung squamous cell carcinoma at 96.43% precision and 93.91% recall. Through our approach, we identified key genes such as C3orf36, JHY, and TASP1, which showed significant differences in mutation counts across various cancer types. Gene enrichment analysis revealed critical pathways like Acute myeloid leukemia and Growth hormone synthesis, secretion and action.

## Materials and methods

### Data accumulation and preparation

**Figure 1:**
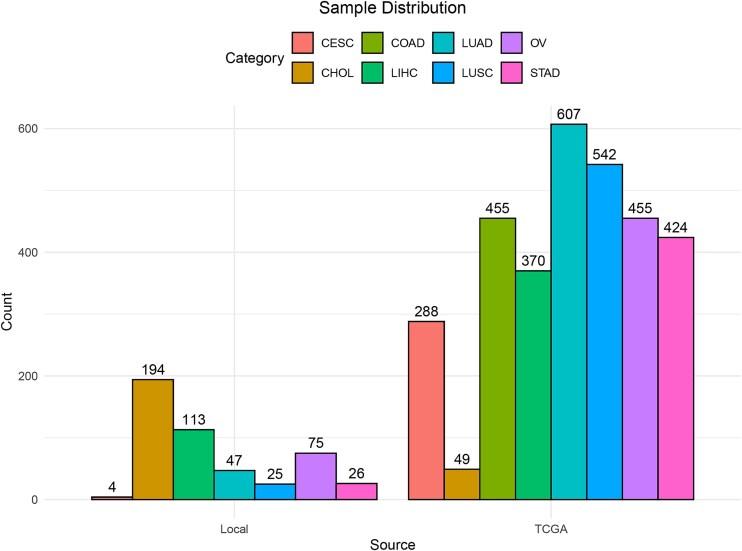
The distribution of samples by source and cancer type. TCGA: Data provided by The Cancer Genome Atlas; Local: Data provided by the authors. CESC: Cervical squamous cell carcinoma and endocervical adenocarcinoma; CHOL: Cholangiocarcinoma; COAD: Colon adenocarcinoma; LIHC: Liver hepatocellular carcinoma; LUAD: Lung adenocarcinoma; LUSC: Lung squamous cell carcinoma; OV: Ovarian serous cystadenocarcinoma; STAD: Stomach adenocarcinoma.

Our data were sourced from somatic mutation data from The Cancer Genome Atlas (TCGA), as well as from local datasets including BGI's 688 Panel sequencing data, whole genome sequencing data, and exome sequencing data (Table 1). We collected mutation annotation format (MAF) files, which contained detailed information about somatic mutations across various cancer types. Specifically, we targeted 8 cancer types, ensuring a comprehensive representation of diverse malignancies. In total, we retrieved data from 3674 samples, including both tumor and adjacent normal tissues. The distribution of samples across cancer types is presented in Fig. 1, with detailed information available in Table S1. To enhance the robustness of our analysis, we filtered out samples lacking primary tumor data and excluded silent mutations to focus solely on non-silent alterations. This curated dataset served as the foundation for subsequent analyses and model development.

**Table 1:** Data sources.

Cancer types	Data types
CESC	exome sequencing data
CHOL	BGI's 688 Panel sequencing data
LIHC	BGI's 688 Panel sequencing data
LUAD	BGI's 688 Panel sequencing data, whole genome sequencing data
LUSC	BGI's 688 Panel sequencing data, whole genome sequencing data
OV	BGI's 688 Panel sequencing data
STAD	exome sequencing data
COAD	\

### Quality control, variant calling and imputation

**Figure 2:**
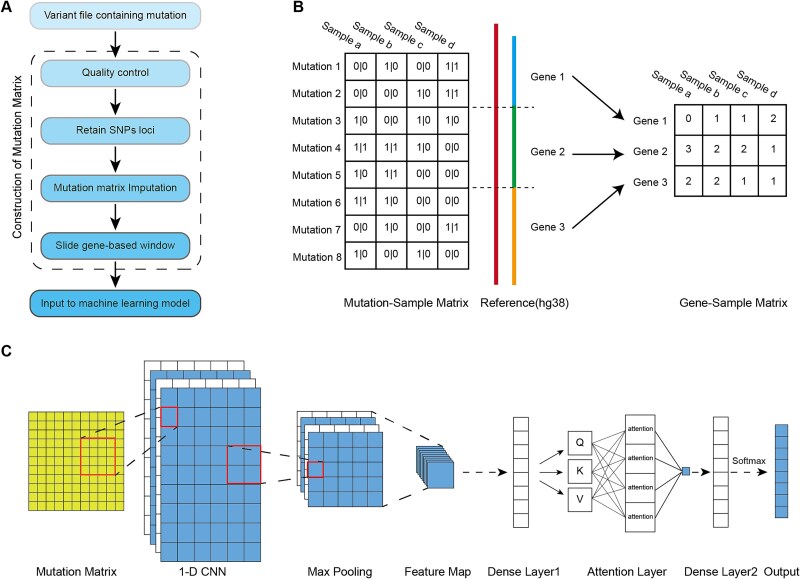
Overview of the cancer classification model. (A) Pipeline for processing mutation data to generate a mutation count matrix; (B) example of annotating mutation files into a mutation count matrix; (C) deep learning architecture.

The sequencing data processing pipeline was meticulously designed to ensure the accuracy and reliability of variant calling and subsequent imputation for our targeted cancer gene panel (Fig. 2A). To standardize genomic data formats for subsequent integrative analysis, we performed systematic conversion of MAF files obtained from The Cancer Genome Atlas (TCGA) to variant call format (VCF) using VCF2MAF (v1.6.17). This conversion procedure ensured compatibility with locally generated VCF files through rigorous format normalization.

Subsequently, we addressed missing variants and enhanced dataset completeness by performing genotype imputation on the consolidated VCF files using BEAGLE 5.5 (version 17Dec24.224). BEAGLE was chosen for its state-of-the-art imputation algorithms, which are particularly effective in handling the complexities of genomic data. This step was crucial in filling gaps in the mutation data from the cancer gene panel, thereby ensuring a robust dataset for downstream analyses.

### Mutation annotation, gene-based feature matrix construction and feature pruning

To precisely map variants identified in our cancer gene panel to their corresponding genomic loci, we employed the RefSeq Release 110 annotation (hg38 assembly), a stable reference resource depicted in Fig. 2B, for comprehensive variant annotation. The annotation process was carried out using the ANNOVAR software (version 2020-06-08), which is renowned for its robust capabilities in variant annotation. ANNOVAR allowed us to map each variant to its respective gene by considering the genomic coordinates and the specific annotation categories provided by RefSeq. This step ensured that each variant was accurately assigned to the appropriate gene, laying the groundwork for the subsequent construction of our mutation feature matrix.

Following the annotation of variants, we constructed a mutation count matrix to encapsulate the number of variants within each gene across all samples in our dataset. The matrix was structured such that each row represented a unique sample $i$, and each column corresponded to a gene $j$. The value at the intersection of row $i$ and column $j$, denoted as ${n}_{ij}$, represented the total number of variants identified in gene $j$ for sample $i$. The construction of the mutation count matrix was executed through the following steps:

(1) For each sample, we iterated through the list of annotated variants and tallied the number of variants associated with each gene. This process was performed using a custom script written in Python.
(2) The resulting counts were organized into a matrix format. Each entry ${n}_{ij}$ in the matrix was calculated by summing the number of variants in gene $j$ for sample $i$. The final matrix was saved as a CSV file to facilitate further analysis and machine learning modeling.

In the initial mutation count matrix, genes (columns) were ordered alphabetically by official gene symbols for standardization and reproducibility, without implying any biological or functional relationship. Prior to model training, genes with low mutation count variance were removed, and the remaining genes were reordered in descending order according to their Fisher scores, such that the final feature sequence reflected statistical discriminative power rather than predefined biological assumptions.

### Self-attention structured machine learning model

To classify cancer subtypes based on the mutation count matrix derived from our cancer gene panel, we developed a deep learning model utilizing PyTorch. The model architecture was designed to integrate convolutional neural networks (CNNs) with attention mechanisms, aiming to enhance classification accuracy and interpretability.

The mutation count matrix served as the input to our model, providing a representation of the mutational landscape across different samples and genes. The model's architecture began with an input layer that fed the mutation count matrix into convolutional layers for feature extraction. These layers were followed by max pooling layers to reduce dimensionality while retaining crucial information. The convolutional layers were succeeded by a self-attention layer, which allowed the model to dynamically weigh the importance of different features, thereby improving its ability to discern significant patterns within the mutation data and enhancing classification performance.

The output from the self-attention layer was then passed through fully connected dense layers. To prevent overfitting and enhance the model's generalization capabilities, dropout was applied to the dense layers. The final layer of the model utilized a softmax activation function, facilitating the classification of samples into distinct cancer subtypes by providing a probability distribution over the possible subtypes.

The entire model was constructed using PyTorch, a deep learning framework that enabled efficient model development and training. The Conv1D and other models’ hyperparameters are detailed in Table S2.

### Training, parameter optimization, and model evaluation

The dataset was divided into a training set (64%), a validation set (16%), and a testing set (20%). This partitioning was designed to ensure robust training, validation, and testing of the model across different subsets of the data.

The model structure is shown in Fig. 2C, it was trained using random weight initialization with a batch size of 256 over 65 epochs. We employed the Adam optimization method with a learning rate of 1e−5 and binary cross-entropy as the loss function. To prevent overfitting, dropout was incorporated into the model architecture. We utilized a grid search approach to optimize the hyperparameters, systematically exploring different combinations to identify the settings that yielded the best performance. The specific hyperparameters and their tuning ranges are detailed in Table S3.

The classification threshold was set to 0.5 by default, indicating that any prediction above this threshold was considered indicative of a specific cancer subtype.

We utilized a confusion matrix derived from the validation dataset to measure the effectiveness of the classification model. From this matrix, we calculated several metrics: accuracy (the percentage of samples correctly predicted), precision (the percentage of predicted positive cases that were correctly predicted), recall (the percentage of actual positive cases that were correctly predicted), and F1-score (the harmonic mean of precision and recall). These metrics provided a comprehensive view of the model's classification performance. We also generated a receiver operating characteristic (ROC) curve to demonstrate the model's performance across all classification thresholds and calculated the area under the ROC curve (AUC) as an aggregate measure of performance.

To estimate the uncertainty of the model, we applied direct bootstrapping through random sampling. We performed 10 000 experiments, calculating the average AUC with a confidence interval for each experiment. Additionally, we measured the correlation coefficient to assess the relationship between the model-predicted values and the true labels, with the *P* calculated using the ‘pearsonr’ function from the Python package ‘scipy’.

### Key genes identified and enrichment

We utilized the self-attention mechanism within our model to derive feature scores for each gene. These feature scores provided a quantitative measure of the importance of each gene in the model's decision-making process. By ranking these scores, we identified the top 1000 genes with the highest impact on the model's performance. These genes were deemed crucial for distinguishing between different cancer subtypes based on their mutation patterns.

To elucidate the biological significance of these high-impact genes, we annotated them to the human genome using RefSeq Release 110. Subsequently, we conducted gene enrichment analyses using both the Kyoto Encyclopedia of Genes and Genomes (KEGG) pathways and Gene Ontology (GO) terms by Metascape (https://metascape.org/). For the enrichment analyses, we employed the R package ‘clusterProfiler.’ To determine statistical significance, we applied the Benjamini-Hochberg correction for multiple testing, setting a threshold of an adjusted *P* < .05 for significance. The KEGG pathway analysis revealed specific biological pathways enriched among the top 1000 genes, providing insights into the molecular mechanisms and signaling pathways potentially altered in the cancer subtypes under study. Similarly, the GO term analysis identified enriched biological processes, molecular functions, and cellular components associated with these genes, allowing us to understand the broader functional categories in which these genes are involved.

### Feature attribution via self-attention mechanism

For each trained model, we extracted attention weights from the self-attention layer for all test samples. Attention weights represent the relative importance assigned by the model to each genomic feature when making predictions. To identify the most influential features per model, we aggregated attention weights across all test samples within each run by computing the mean attention weight for each feature. Features were then ranked in descending order, and the top 1000 highest-weighted features were selected as the model's interpretable feature set for that run.

This procedure was repeated independently for all trained models, yielding 1000 sets of top-ranked features. To systematically quantify feature selection consistency across runs, we constructed a binary presence-absence matrix where rows represent individual model runs (n = 1000) and columns represent unique genomic features identified across all runs (n = 44 607 total features in the input data). A matrix entry was coded as 1 if a given feature appeared in the top 1000 for that specific run, and 0 otherwise. This representation enabled calculation of the selection frequency for each feature, defined as the proportion of runs in which the feature was selected.

### Statistical identification of stably selected features

To distinguish features selected more frequently than expected by random chance, we applied a binomial test to each feature's selection frequency. Under the null hypothesis of random selection without biological signal, the probability that any given feature is selected in a single run is p_0_ = k/N, where k = 1000 (number of selected features per run) and N = 44,607 (total feature pool size). For each feature i with observed selection count x_i across n = 1000 runs, we computed the one-sided binomial test *P*-value as:


$$ \mathrm{P}\left(\mathrm{X}\ge \mathrm{x}\_\mathrm{i}|\mathrm{n}=1000,{\mathrm{p}}_0=1000/44607\right) $$

This tests whether the observed selection frequency significantly exceeds the random expectation. To control the false discovery rate arising from testing 44 607 features simultaneously, we applied Benjamini-Hochberg FDR correction using the multipletests() function from the statsmodels package (v0.14.2). Features with FDR-corrected q-values <0.05 were classified as significantly stable.

All stability analyses were implemented in Python 3.10.14 using NumPy (v1.26.4) for numerical operations, SciPy (v1.13.1) for statistical testing, pandas (v1.5.3) for data manipulation, and statsmodels (v0.14.2) for multiple testing correction. Visualizations were generated using Matplotlib (v3.8.4) and Seaborn (v0.13.2).

### Gene annotation and functional enrichment analysis

To assess the biological relevance of stably selected features, we performed functional enrichment analysis on features with high selection frequency (FDR< 0.05). Each genomic feature index was first mapped to its corresponding gene symbol(s) based on genomic coordinates. For features spanning multiple genes or gene boundaries, all associated gene symbols were extracted. When a feature's annotation contained multiple genes separated by semicolons (encoded as \x3b in the data), we parsed and de-duplicated all symbols to create a comprehensive gene list.

Gene symbols were converted to Entrez Gene IDs using the bitr() function from the clusterProfiler R package (v4.10.1), querying the org.Hs.eg.db annotation database (v3.18.0) for *Homo sapiens*. We performed Gene Ontology (GO) enrichment analysis across all three ontologies—Biological Process.

## Results

### High accuracy of self-attention based Conv1D network in classifying variant cancer types

**Figure 3:**
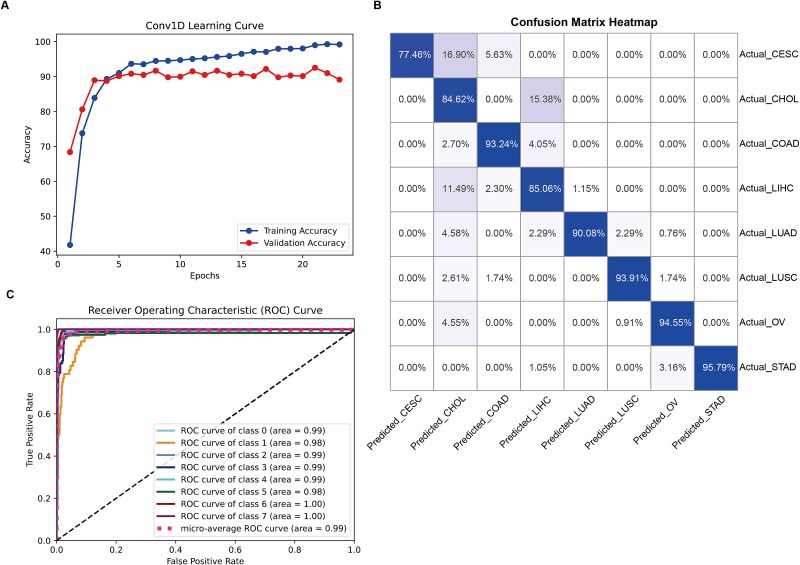
Performance of the deep learning model. (A) Learning curves for the training and testing sets; (B) confusion matrix for model predictions across eight cancer types; (C) ROC curves. Class 0: CESC; class 1: CHOL; class 2: COAD; class 3: LIHC; class 4: LUAD; class 5: LUSC; class 6: OV; class 7: STAD.

Our study focused on developing a self-attention based Conv1D machine learning network to enhance cancer classification accuracy using genomic variant data. This approach demonstrated remarkable performance in classifying various cancer types, as evidenced by our analysis of the learning curve, learning result statistics, and the Receiver Operating Characteristic (ROC) curve. The learning curve, depicted in Fig. 3A, showed the model's accuracy over 23 epochs. Initially, the training accuracy rapidly increased from ~40% to ~85% within the first few epochs, indicating quick learning of fundamental patterns. The validation accuracy followed a similar trend, starting at ~70% and reaching 90% by the 5th epoch, suggesting effective generalization to unseen data. As training progressed, both training and validation accuracies continued to improve, with training accuracy remained at or above 95% after the 10th epoch, while validation accuracy reached around 90% by the 15th epoch. This convergence at high levels indicated successful classification without overfitting. The final epochs showed minimal fluctuations, with training accuracy just above 95% and validation accuracy maintaining a steady 90%, demonstrating the model's robustness and ability to maintain high accuracy.

Our model achieved an overall classification accuracy of over 90%, as detailed in the learning result statistics, showcasing its effectiveness in distinguishing between various cancer types. The confusion matrix revealed high precision and recall across most cancer types (Table 2). For instance, lung squamous cell carcinoma was classified with a precision of 96.43% and a recall of 93.91%, indicating minimal misclassifications. Similarly, colon cancer showed a precision of 89.61% and a recall of 93.24%. However, it should be noted that certain malignancies exhibit lower diagnostic accuracy, which may be attributed to substantial genetic overlap with other cancer types that share common molecular signatures and pathogenic pathways. For example, cholangiocarcinoma and cervical cancer exhibited some overlap in classification, with a few instances of cervical cancer being misclassified as cholangiocarcinoma and vice versa. Despite these minor errors, the overall performance remained exceptionally high, with most cancer types achieving precision and recall rates around 90%. This high level of accuracy underscores the potential of our self-attention based Conv1D network in clinical settings. The model's ability to handle the complexity of genomic data was highlighted, effectively capturing nuanced patterns that distinguish one cancer type from another, with the attention mechanism playing a pivotal role in focusing on the most relevant genetic variants.

**Table 2:** Precision, recall and f1-score across cancer types.

Class	Precision	Recall	F1-score
CESC	1	0.7746	0.873
CHOL	0.5366	0.8462	0.6567
COAD	0.8961	0.9324	0.9139
LIHC	0.8315	0.8506	0.8409
LUAD	0.9916	0.9008	0.944
LUSC	0.9643	0.9391	0.9515
OV	0.9455	0.9455	0.9455
STAD	1	0.9579	0.9785

The confusion matrix heatmap (Fig. 3B) clearly illustrates the model's performance across eight different cancer types: cervical squamous cell carcinoma and endocervical adenocarcinoma (CESC), cholangiocarcinoma (CHOL), colon adenocarcinoma (COAD), liver hepatocellular carcinoma (LIHC), lung adenocarcinoma (LUAD), lung squamous cell carcinoma (LUSC), ovarian serous cystadenocarcinoma (OV), and stomach adenocarcinoma (STAD). The diagonal elements of the matrix show the correct classification rates, with CESC achieving 77.46%, CHOL at 84.62%, COAD at 93.24%, LIHC at 85.06%, LUAD at 90.08%, LUSC at 93.91%, OV at 94.55%, and STAD at 95.79%. These high values along the diagonal indicate the model's strong ability to correctly classify each cancer type. Off-diagonal elements represent misclassifications, with the most notable being 16.90% of CESC samples misclassified as CHOL and 15.38% of CHOL samples misclassified as COAD.

The ROC curve, as depicted in Fig. 3C provided another valuable perspective on the model's performance. The ROC curve illustrates the trade-off between the true positive rate (sensitivity) and the false positive rate (1-specificity) for different classification thresholds. The area under the curve (AUC) values for individual cancer classes ranged from 0.98 to 1.00, indicating excellent discrimination ability. Specifically, the AUC for class 0 was 0.99, class 1 was 0.98, class 2 was 0.99, class 3 was 0.99, class 4 was 0.99, class 5 was 0.98, class 6 was 1.00, and class 7 was 1.00. The micro-average ROC curve, which aggregates the performance across all classes, achieved an AUC of 0.99, further validating the high overall performance of our model. The ROC curves demonstrated that our model maintained high sensitivity and specificity across all cancer types, with minimal false positives and high true positives. This robust performance across different classes underscored the model's ability to accurately classify cancer types while minimizing misclassifications.

### Genetic proximity of cancer types revealed through distance matrix and hierarchical clustering

**Figure 4:**
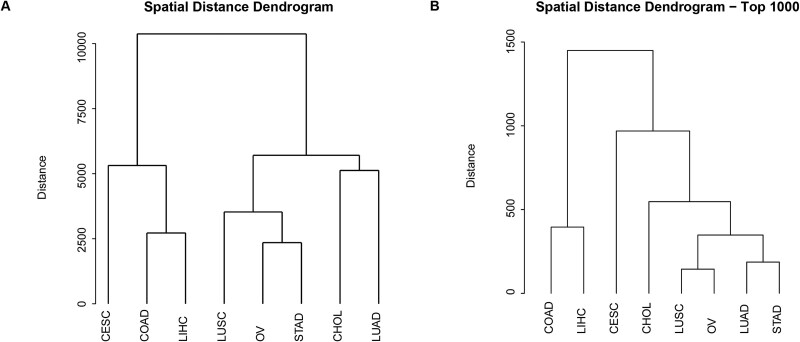
Spatial distance dendrogram of cancer types. (A) Complete linkage spatial distance dendrogram based on all genes; (B) complete linkage spatial distance dendrogram based on the top 1000 genes.

We employed hierarchical clustering to explore the genetic proximity of different cancer types, utilizing both a comprehensive dataset encompassing all genes and a focused dataset consisting of the top 1000 genes identified through our self-attention based Conv1D network. This analysis aimed to uncover underlying genetic relationships between cancer types that may not be apparent through traditional clinical or organ-based classifications. The first dendrogram, depicted in Fig. 4A was constructed using all available genes. The spatial distance dendrogram, using complete linkage and Euclidean distance, reveals the hierarchical clustering of these cancer types based on their genetic profiles. In this dendrogram, the maximum spatial distance reaches up to 10 000 units, indicating significant genetic variation between the cancer types. The tree shows that COAD and LIHC cluster together at a relatively low distance, suggesting a closer genetic relationship between these two cancer types. Similarly, LUSC and OV also form a cluster, indicating shared genetic characteristics. On the other hand, CHOL and LUAD appear to be more genetically distant from the other cancer types, with CHOL branching off early in the dendrogram.

The second dendrogram, depicted in Fig. 4B was constructed using only the top 1000 genes identified by the attention layer in our self-attention based Conv1D network. This focused approach aims to highlight the genetic proximity of cancer types based on the most influential genes in our classification model. In this dendrogram, the maximum spatial distance is significantly reduced to 1500 units, indicating that the genetic variation captured by the top 1000 genes is less than that captured by all genes. The tree reveals a different clustering pattern compared to the all-genes dendrogram. Here, COAD and LIHC still cluster together, but at a much closer distance, reinforcing their genetic similarity. Interestingly, CESC and CHOL now form a distinct cluster, suggesting that these cancer types share significant genetic features when considering only the top 1000 genes. LUSC and OV, which clustered together in the all-genes dendrogram, are now more distant from each other, with LUSC aligning more closely with LUAD and STAD.

### Identification of key genes in cancer classification using attention layer

The self-attention based Conv1D network played a crucial role in identifying key genes that significantly contribute to the classification of various cancer types. By leveraging the attention mechanism, our model focused on the most relevant genetic variants, enhancing its classification accuracy. We analyzed box plots for the top 10 genes with the largest intergroup differences from the top 50 weighted genes, and conducted KEGG and GO pathway enrichment analysis on the top 1000 genes to understand the biological pathways associated with these genes.

**Figure 5:**
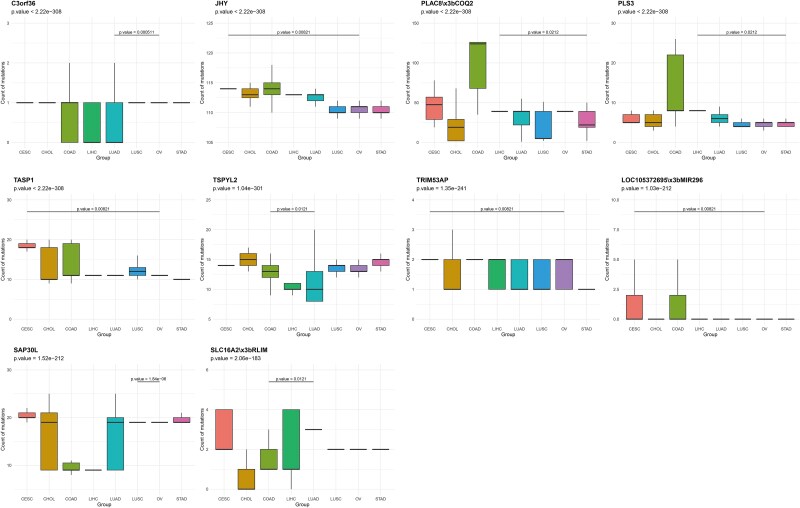
Boxplots of the top 10 genes with the lowest Kruskal-Wallis test Ps among the top 50 genes by weight.

The box plots, depicted in Fig. 5 visually represent the distribution of mutation counts across different cancer types for genes such as *C3orf36*, *JHY*, *PLAC8*;*COQ2*, *PLS3*, *TASP1*, *TSPYL2*, *TRIM53AP*, LOC105372695;MIR296, *SAP30L*, and *SLC16A2*;*RLIM*, allowing us to assess the variability and significance of mutations in each gene across cancer types. For *C3orf36*, the box plot showed a significant difference in mutation counts across cancer types with Kruskal-Wallis *P* < 2.22e-308, indicating strong statistical significance. The median mutation count varied from 0 to 7.5, with CESC showing the highest median mutation count. Similarly, *JHY* exhibited significant differences in mutation counts, with Kruskal-Wallis *P <* 2.22e-308. The median mutation count ranged from 120 to 160, with COAD showing the highest median mutation count. *PLAC8*;*COQ2* and *PLS3* also showed significant differences in mutation counts across cancer types, with Kruskal-Wallis *P <* 2.22e-308. The median mutation counts for *PLAC8;COQ2* ranged from 0 to 100, while for *PLS3*, they ranged from 10 to 40. *TASP1*, with Kruskal-Wallis *P <* 2.22e-308, showed median mutation counts ranging from 0 to 250, with *LIHC* having the highest median count. *TSPYL2*, *TRIM53AP*, LOC105372695;MIR296, *SAP30L*, and *SLC16A2*;*RLIM* also exhibited significant differences in mutation counts across cancer types, with Kruskal-Wallis *P* ranging from 1.04e-301 to 2.06e-183. These genes showed varying median mutation counts, with some cancer types consistently showing higher mutation counts than others, indicating their potential role in cancer classification and development.

**Figure 6:**
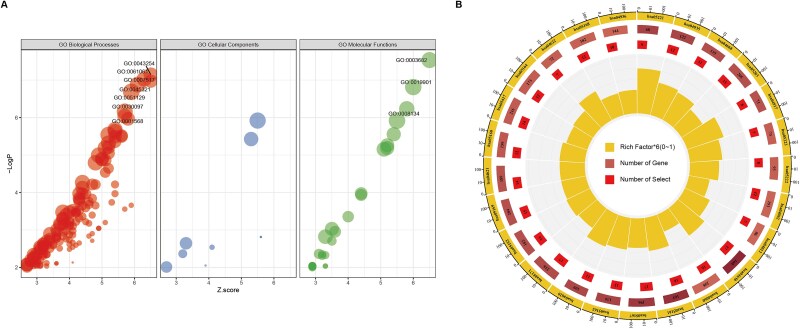
Enrichment plots of the top 1000 genes by weight. (A) GO enrichment analysis; (B) KEGG pathway enrichment.

To identify key pathways involved in cancer type differentiation, we analyzed the top 1000 genes contributing to classification across eight cancer types (Table S4). We subsequently performed Gene Ontology (GO) enrichment analysis on these genes to elucidate the biological processes, molecular functions, and cellular components most relevant to each cancer type (Table 3). GO enrichment analysis revealed several significantly enriched pathways (Fig. 6A). Chromatin binding (GO:0003682) emerged as the most significantly enriched molecular function (LogP = −7.53), highlighting the critical role of epigenetic regulation in cancer type specificity. Cytoskeletal regulation pathways were prominently enriched, particularly regulation of protein-containing complex assembly (GO:0043254, LogP = −7.14) and microtubule-associated processes. These findings align with the established importance of cytoskeletal dynamics in cancer migration and invasion mechanisms. Tissue-specific developmental pathways were markedly enriched, including muscle structure development (GO:0061061, LogP = −7.03) and heart development (GO:0007507), suggesting developmental program reactivation as a distinguishing feature between cancer types. Immune-related processes, particularly leukocyte activation (GO:0045321, LogP = −6.88) and hemopoiesis (GO:0030097, LogP = −6.16), were significantly enriched, reflecting cancer type-specific immune microenvironment signatures. Angiogenesis (GO:0001525, LogP = −4.86) and vasculature development (GO:0001944) pathways demonstrated high enrichment scores, consistent with the varied vascularization patterns observed across different cancer types. 

**Table 3:** GO enrichment analysis result.

GroupID	Category	ID	Description	LogP	Log.q.value.	Genes	Symbols	InTerm_InList	Count	Z.score
1_Member	GO Molecular Functions	GO:0003682	chromatin binding	−7.5347	−3.13	#####	CCNT2,CENPB,CHD2,CHD3,DMRT1,ELK1,EP300,FOS,H1–2,H1–4,HOXD13,MNT,PAX6,SFPQ,SHMT2,STAT3,STAT5B,TAL1,TOP2A,TOP2B,TTF1,NELFA,KAT2B,SETDB1,STAG3,PSIP1,BAHD1,KDM1A,PHF8,KAT6B,NUP62,PELP1,ZNF304,CHD8,TSPYL2,NOC3L,SAP30L,ZNF750,CBX2,PWWP3A,MBD6,ARX,ATXN1L	43/615	43	6.5
2_Member	GO Biological Processes	GO:0043254	regulation of protein-containing complex assembly	−7.1443	−3.13	#####	ADD2,RHOC,CDH5,EP300,HSPA5,LGALS3,MAP1B,PIK3R2,PPP2R5B,PTGER4,SVIL,TAL1,TBCD,NAPA,TNFSF18,CKAP5,FARP2,SEC16A,HAX1,UNC13B,ARFGEF1,CLASP2,CAMSAP2,TRAPPC12,CRBN,ASB2,HAUS6,SPTBN4,ZDHHC12,NAV3,ARHGAP18,TIRAP,TPPP2,NEK7	34/438	34	6.5
2_Member	GO Biological Processes	GO:0043242	negative regulation of protein-containing complex disassembly	−4.2148	−1.62	#####	ADD2,MAP1B,SVIL,RUBCN,CLASP2,CAMSAP2,CKAP2,SCAF4,SPTBN4,NAV3	10/79	10	5.4
2_Member	GO Biological Processes	GO:0032271	regulation of protein polymerization	−4.213	−1.62	#####	ADD2,CDH5,MAP1B,PIK3R2,SVIL,TBCD,CKAP5,HAX1,ARFGEF1,CLASP2,CAMSAP2,ASB2,HAUS6,SPTBN4,NAV3,ARHGAP18,TPPP2	17/206	17	4.8
2_Member	GO Biological Processes	GO:0051493	regulation of cytoskeleton organization	−3.9688	−1.48	#####	ADD2,RHOC,CDH5,MAP1B,NEB,PIK3R1,PIK3R2,PTGER4,PTK2,SLC4A2,SVIL,TBCD,KAT2B,TAOK2,CKAP5,HAX1,ARFGEF1,CLASP2,CAMSAP2,NUP62,CKAP2,STAU2,ASB2,HAUS6,SPTBN4,NAV3,FRMD7,MYLK3,ARHGAP18,SKA3,MIR335	31/539	31	4.3
2_Member	GO Biological Processes	GO:0051494	negative regulation of cytoskeleton organization	−3.7557	−1.35	#####	ADD2,CDH5,MAP1B,PIK3R1,SVIL,TBCD,KAT2B,ARFGEF1,CLASP2,CAMSAP2,CKAP2,SPTBN4,NAV3,FRMD7	14/163	14	4.6
2_Member	GO Biological Processes	GO:0031111	negative regulation of microtubule polymerization or depolymerization	−3.7368	−1.34	#####	CDH5,MAP1B,TBCD,CLASP2,CAMSAP2,CKAP2,NAV3	7/44	7	5.3
2_Member	GO Biological Processes	GO:0043244	regulation of protein-containing complex disassembly	−3.5889	−1.26	#####	ADD2,MAP1B,SVIL,RUBCN,CLASP2,CAMSAP2,TECPR1,CKAP2,ASB2,SCAF4,SPTBN4,NAV3	12/130	12	4.5
2_Member	GO Biological Processes	GO:0010639	negative regulation of organelle organization	−3.5376	−1.23	#####	ADD2,CDH5,DMRT1,LMNA,MAP1B,PIK3R1,RAD1,SVIL,TBCD,TOP2A,KAT2B,USP10,KNTC1,ARFGEF1,CLASP2,CAMSAP2,CKAP2,RTEL1,TEX14,SPTBN4,NAV3,FRMD7	22/348	22	4.1
2_Member	GO Biological Processes	GO:0031113	regulation of microtubule polymerization	−3.3974	−1.16	#####	CDH5,MAP1B,TBCD,CKAP5,CLASP2,CAMSAP2,HAUS6,NAV3	8/65	8	4.7
2_Member	GO Biological Processes	GO:0031110	regulation of microtubule polymerization or depolymerization	−3.3593	−1.14	#####	CDH5,MAP1B,TBCD,CKAP5,CLASP2,CAMSAP2,CKAP2,HAUS6,NAV3,SKA3	10/100	10	4.4
2_Member	GO Biological Processes	GO:1901880	negative regulation of protein depolymerization	−3.22	−1.06	#####	ADD2,MAP1B,SVIL,CLASP2,CAMSAP2,CKAP2,SPTBN4,NAV3	8/69	8	4.5
2_Member	GO Biological Processes	GO:1902904	negative regulation of supramolecular fiber organization	−3.0972	−1	#####	ADD2,CDH5,MAP1B,PIK3R1,SVIL,TBCD,ARFGEF1,CLASP2,CAMSAP2,CKAP2,SPTBN4,NAV3,FRMD7	13/168	13	4
2_Member	GO Biological Processes	GO:1901879	regulation of protein depolymerization	−2.9755	−0.94	#####	ADD2,MAP1B,SVIL,CLASP2,CAMSAP2,CKAP2,ASB2,SPTBN4,NAV3	9/93	9	4.1
2_Member	GO Molecular Functions	GO:0015631	tubulin binding	−2.9541	−0.93	#####	ALDOA,DCX,DYNC1I1,FNTA,MAP1B,TBCD,CKAP5,DNM1L,RGS14,CLASP2,SYT11,CAMSAP2,SETD2,SBDS,GTSE1,HAUS6,TTLL2,MAP1LC3A,NAV3,TPPP2,SKA3,MAP1LC3B2	22/385	22	3.6
2_Member	GO Biological Processes	GO:0007026	negative regulation of microtubule depolymerization	−2.8358	−0.85	#####	MAP1B,CLASP2,CAMSAP2,CKAP2,NAV3	#######	5	4.6
2_Member	GO Biological Processes	GO:0032970	regulation of actin filament-based process	−2.772	−0.83	#####	ADD2,RHOC,DSP,MYH9,NEB,PIK3R1,PIK3R2,PTGER4,SLC4A2,STC1,SVIL,TAOK2,HAX1,ARFGEF1,CLASP2,STAU2,ASB2,SPTBN4,FRMD7,MYLK3,ARHGAP18,MIR335	22/398	22	3.4
2_Member	GO Biological Processes	GO:1902903	regulation of supramolecular fiber organization	−2.7181	−0.8	#####	ADD2,RHOC,CDH5,MAP1B,PIK3R1,PIK3R2,PTGER4,SVIL,TBCD,CKAP5,HAX1,ARFGEF1,CLASP2,CAMSAP2,CKAP2,ASB2,HAUS6,SPTBN4,NAV3,FRMD7,MYLK3,ARHGAP18	22/402	22	3.4
2_Member	GO Cellular Components	GO:0005874	microtubule	−2.6442	−0.77	#####	DCX,DYNC1I1,DPYSL2,MAP1B,SVIL,TBCD,KNTC1,CKAP5,DNM1L,RGS14,CLASP2,CAMSAP2,CKAP2,STAU2,GTSE1,HAUS6,MAP1LC3A,NAV3,ARHGAP18,TPPP2,NEK7,TTLL8,SKA3,CFAP77,MAP1LC3B2	25/486	25	3.3
2_Member	GO Biological Processes	GO:0031333	negative regulation of protein-containing complex assembly	−2.615	−0.75	#####	ADD2,CDH5,EP300,HSPA5,PTGER4,SVIL,TBCD,ARFGEF1,CRBN,SPTBN4,ZDHHC12	11/146	11	3.5
2_Member	GO Cellular Components	GO:1990752	microtubule end	−2.5388	−0.71	#####	SVIL,CKAP5,CLASP2,CAMSAP2,NAV3	5/36	5	4.1
2_Member	GO Biological Processes	GO:0031114	regulation of microtubule depolymerization	−2.4855	−0.67	#####	MAP1B,CLASP2,CAMSAP2,CKAP2,NAV3	5/37	5	4
2_Member	GO Biological Processes	GO:0032886	regulation of microtubule-based process	−2.469	−0.67	#####	CDH5,LAMP1,MAP1B,TBCD,KAT2B,CKAP5,PDCD6IP,CLASP2,CAMSAP2,NUP62,CKAP2,HAUS6,NAV3,TPPP2,SKA3,CCDC39	16/269	16	3.2
2_Member	GO Molecular Functions	GO:0008017	microtubule binding	−2.3615	−0.6	#####	DCX,DYNC1I1,FNTA,MAP1B,CKAP5,DNM1L,RGS14,CLASP2,CAMSAP2,SBDS,GTSE1,HAUS6,MAP1LC3A,NAV3,SKA3,MAP1LC3B2	16/276	16	3.1
2_Member	GO Biological Processes	GO:0032956	regulation of actin cytoskeleton organization	−2.3345	−0.59	#####	ADD2,RHOC,NEB,PIK3R1,PIK3R2,PTGER4,SLC4A2,SVIL,TAOK2,HAX1,ARFGEF1,CLASP2,STAU2,ASB2,SPTBN4,FRMD7,MYLK3,ARHGAP18,MIR335	19/354	19	3
2_Member	GO Biological Processes	GO:0070507	regulation of microtubule cytoskeleton organization	−2.1934	−0.52	#####	CDH5,MAP1B,TBCD,CKAP5,CLASP2,CAMSAP2,NUP62,CKAP2,HAUS6,NAV3,SKA3	11/166	11	3.1
3_Member	GO Biological Processes	GO:0061061	muscle structure development	−7.0282	−3.13	34,89,104,607,905,1106,1271,1832,1960,2033,2353,3911,4000,4209,4627,4638,4703,5239,5915,6442,6608,6840,7291,7402,10,529,23,028,51,676,57,462,60,529,64,091,64,208,79,784,80,005,84,466,85,407,91,807,219,537,283,078	ACADM,ACTN3,ADARB1,BCL9,CCNT2,CHD2,CNTFR,DSP,EGR3,EP300,FOS,LAMA5,LMNA,MEF2D,MYH9,MYLK,NEB,PGM5,RARB,SGCA,SMO,SVIL,TWIST1,UTRN,NEBL,KDM1A,ASB2,MYORG,ALX4,POPDC2,POPDC3,MYH14,DOCK5,MEGF10,NKD1,MYLK3,SMTNL1,MKX	38/529	38	6.3
3_Member	GO Biological Processes	GO:0007517	muscle organ development	−6.9707	−3.13	#####	ADARB1,BCL9,CCNT2,CHD2,CNTFR,DSP,EGR3,EP300,FOS,LAMA5,LMNA,MEF2D,MYLK,NEB,SGCA,SMO,SVIL,TWIST1,UTRN,ASB2,MYORG,ALX4,POPDC2,POPDC3,MYH14,MEGF10,SMTNL1,MKX	28/323	28	6.5
3_Member	GO Biological Processes	GO:0007507	heart development	−4.1248	−1.55	#####	ACADM,CASP8,COL5A1,CRKL,DSP,MEGF8,EP300,EPHB4,FOLR1,LMNA,MEF2D,NEB,NFATC1,NTRK3,PPARD,PTK2,PTPRJ,RARB,SMO,TWIST1,KAT2B,CACNA1G,CALCRL,NEBL,ADAMTS6,SETD2,ASB2,POPDC2,POPDC3,MYLK3,ANKS6,SH3PXD2B,CCDC39	33/578	33	4.4
3_Member	GO Biological Processes	GO:0060538	skeletal muscle organ development	−3.8407	−1.42	#####	BCL9,CCNT2,CNTFR,EP300,FOS,MEF2D,SMO,SVIL,ASB2,MYORG,POPDC2,POPDC3,MYH14,MEGF10	14/160	14	4.7
3_Member	GO Biological Processes	GO:0060537	muscle tissue development	−3.8077	−1.39	#####	ACADM,BCL9,CCNT2,DSP,EP300,FOS,LMNA,MEF2D,MYLK,NEB,PGM5,RARB,SMO,SVIL,CACNA1G,NEBL,ASB2,MYORG,POPDC2,POPDC3,MYH14,MEGF10,MYLK3	23/356	23	4.3
3_Member	GO Biological Processes	GO:0014706	striated muscle tissue development	−3.7684	−1.36	#####	ACADM,BCL9,CCNT2,DSP,EP300,FOS,LMNA,MEF2D,NEB,PGM5,RARB,SMO,SVIL,CACNA1G,NEBL,ASB2,MYORG,POPDC2,POPDC3,MYH14,MEGF10,MYLK3	22/335	22	4.3
3_Member	GO Biological Processes	GO:0007519	skeletal muscle tissue development	−3.6832	−1.31	#####	BCL9,CCNT2,EP300,FOS,MEF2D,SMO,SVIL,ASB2,MYORG,POPDC2,POPDC3,MYH14,MEGF10	13/146	13	4.6
3_Member	GO Biological Processes	GO:0042692	muscle cell differentiation	−2.9457	−0.93	34,89,607,4000,4627,4703,5239,5915,6608,10,529,23,028,51,676,57,462,64,091,64,208,80,005,84,466,91,807	ACADM,ACTN3,BCL9,LMNA,MYH9,NEB,PGM5,RARB,SMO,NEBL,KDM1A,ASB2,MYORG,POPDC2,POPDC3,DOCK5,MEGF10,MYLK3	18/288	18	3.6
3_Member	GO Biological Processes	GO:0051146	striated muscle cell differentiation	−2.8388	−0.85	#####	ACADM,BCL9,LMNA,MYH9,NEB,PGM5,RARB,SMO,NEBL,ASB2,MYORG,POPDC2,POPDC3,DOCK5,MYLK3	15/224	15	3.6
3_Member	GO Biological Processes	GO:0035051	cardiocyte differentiation	−2.0873	−0.46	#####	ACADM,FOLR1,LMNA,NEB,RARB,TWIST1,NEBL,ASB2,MYLK3	9/126	9	3
3_Member	GO Biological Processes	GO:0055001	muscle cell development	−2.0641	−0.45	#####	ACTN3,LMNA,NEB,PGM5,SMO,NEBL,KDM1A,ASB2,MYORG,MEGF10,MYLK3	11/173	11	2.9
4_Member	GO Biological Processes	GO:0045321	leukocyte activation	−6.8807	−3.13	#####	ANXA3,B2M,C3,C5AR1,CASP8,CD74,DDOST,DNASE1L3,EP300,IFNA10,IFNA14,IFNGR1,IL4R,LBP,MYH9,PIK3CG,PIK3R1,PIK3R2,PTGDR,PTGER4,PTPRC,PTPRJ,STAT3,STAT5B,TOP2B,FZD5,NR4A3,DYSF,TNFSF18,MAFB,CCR9,GPR89B,DOCK10,KMT2E,IFNK,WNK4,TNFAIP8L2,SEMA6D,HSH2D,ADGRG3,THEMIS,NRARP	42/626	42	6.1
4_Member	GO Biological Processes	GO:0030097	hemopoiesis	−6.1615	−2.7	#####	ADD2,B2M,CASP8,CD74,CHD2,EP300,FOS,IL4R,JUNB,LBR,MYH9,NFATC1,PIK3R1,PIK3R2,PRTN3,PTGER4,PTPRC,PTPRJ,SLC4A2,STAT3,STAT5B,TAL1,TOP2A,TOP2B,ZBTB16,FZD5,DYRK3,SOCS1,KRT75,FARP2,MAFB,CCR9,NBEAL2,SBDS,GPR89B,DOCK10,SLC48A1,KMT2E,TIRAP,ADGRG3,HEATR9,THEMIS,NRARP	43/690	43	5.7
4_Member	GO Biological Processes	GO:0002521	leukocyte differentiation	−5.9679	−2.63	#####	B2M,CASP8,CD74,EP300,FOS,IL4R,JUNB,LBR,MYH9,NFATC1,PIK3R1,PIK3R2,PRTN3,PTGER4,PTPRC,PTPRJ,SLC4A2,STAT3,STAT5B,TAL1,TOP2B,FZD5,SOCS1,FARP2,MAFB,CCR9,GPR89B,DOCK10,ADGRG3,THEMIS,NRARP	31/426	31	5.8
4_Member	GO Biological Processes	GO:0001775	cell activation	−5.539	−2.39	#####	ANXA3,B2M,C3,C5AR1,CASP8,CD74,DDOST,DNASE1L3,EP300,IFNA10,IFNA14,IFNGR1,IL4R,LBP,MYH9,PIK3CG,PIK3R1,PIK3R2,PTGDR,PTGER4,PTPRC,PTPRJ,SMO,STAT3,STAT5B,TOP2B,FZD5,NR4A3,DYSF,TNFSF18,MAFB,CCR9,GPR89B,DOCK10,KMT2E,IFNK,WNK4,TNFAIP8L2,SEMA6D,MEGF10,HSH2D,ADGRG3,THEMIS,NRARP	44/754	44	5.2
4_Member	GO Biological Processes	GO:0042110	T cell activation	−5.5184	−2.39	#####	B2M,CASP8,CD74,DDOST,IFNA10,IFNA14,IL4R,MYH9,PIK3CG,PIK3R1,PIK3R2,PTGER4,PTPRC,STAT3,STAT5B,FZD5,TNFSF18,MAFB,CCR9,GPR89B,IFNK,TNFAIP8L2,SEMA6D,HSH2D,THEMIS,NRARP	26/339	26	5.6
4_Member	GO Biological Processes	GO:1903131	mononuclear cell differentiation	−5.05	−2.18	#####	B2M,CASP8,CD74,EP300,FOS,IL4R,MYH9,NFATC1,PIK3R1,PIK3R2,PRTN3,PTGER4,PTPRC,PTPRJ,STAT3,STAT5B,TOP2B,FZD5,SOCS1,MAFB,CCR9,GPR89B,DOCK10,ADGRG3,THEMIS,NRARP	26/360	26	5.2
4_Member	GO Biological Processes	GO:0046649	lymphocyte activation	−4.9036	−2.1	#####	B2M,C3,CASP8,CD74,DDOST,EP300,IFNA10,IFNA14,IL4R,MYH9,PIK3CG,PIK3R1,PIK3R2,PTGER4,PTPRC,PTPRJ,STAT3,STAT5B,TOP2B,FZD5,TNFSF18,MAFB,CCR9,GPR89B,DOCK10,IFNK,TNFAIP8L2,SEMA6D,HSH2D,ADGRG3,THEMIS,NRARP	32/504	32	5
4_Member	GO Biological Processes	GO:0002366	leukocyte activation involved in immune response	−4.6437	−1.9	#####	ANXA3,CD74,DNASE1L3,IFNA10,IFNA14,IL4R,LBP,PIK3CG,PIK3R1,PTGDR,PTGER4,STAT3,NR4A3,DYSF,TNFSF18,DOCK10,IFNK,SEMA6D	18/210	18	5.2
4_Member	GO Biological Processes	GO:0002263	cell activation involved in immune response	−4.5343	−1.8	#####	ANXA3,CD74,DNASE1L3,IFNA10,IFNA14,IL4R,LBP,PIK3CG,PIK3R1,PTGDR,PTGER4,STAT3,NR4A3,DYSF,TNFSF18,DOCK10,IFNK,SEMA6D	18/214	18	5.1
4_Member	GO Biological Processes	GO:0030098	lymphocyte differentiation	−3.9633	−1.48	#####	B2M,CD74,EP300,IL4R,PIK3R1,PIK3R2,PTGER4,PTPRC,PTPRJ,STAT3,STAT5B,TOP2B,FZD5,MAFB,CCR9,GPR89B,DOCK10,ADGRG3,THEMIS,NRARP	20/280	20	4.5
4_Member	GO Biological Processes	GO:0002286	T cell activation involved in immune response	−3.6543	−1.3	#####	CD74,IFNA10,IFNA14,IL4R,PIK3R1,PTGER4,STAT3,TNFSF18,IFNK,SEMA6D	10/92	10	4.8
4_Member	GO Biological Processes	GO:0030217	T cell differentiation	−3.6533	−1.3	#####	B2M,CD74,IL4R,PIK3R1,PIK3R2,PTGER4,PTPRC,STAT3,STAT5B,FZD5,MAFB,CCR9,GPR89B,THEMIS,NRARP	15/187	15	4.4
4_Member	GO Biological Processes	GO:0002252	immune effector process	−3.121	−1.01	#####	ANXA3,B2M,C3,CD74,CD55,DNASE1L3,IFNA10,IFNA14,IL4R,LBP,PIK3CG,PIK3R1,PTGDR,PTGER4,PTK2,STAT3,STAT5B,NR4A3,DYSF,TNFSF18,C1RL,DOCK10,KMT2E,IFNK,SEMA6D,SFTPA1	26/474	26	3.7
4_Member	GO Biological Processes	GO:0042113	B cell activation	−2.951	−0.93	#####	C3,CASP8,EP300,IFNA10,IFNA14,PIK3R1,PIK3R2,PTPRC,PTPRJ,STAT5B,TOP2B,DOCK10,IFNK,ADGRG3	14/196	14	3.8
4_Member	GO Biological Processes	GO:0002285	lymphocyte activation involved in immune response	−2.5918	−0.74	#####	CD74,IFNA10,IFNA14,IL4R,PIK3R1,PTGER4,STAT3,TNFSF18,DOCK10,IFNK,SEMA6D	11/147	11	3.5
4_Member	GO Biological Processes	GO:0030183	B cell differentiation	−2.3421	−0.59	#####	EP300,PIK3R1,PIK3R2,PTPRC,PTPRJ,STAT5B,TOP2B,DOCK10,ADGRG3	9/115	9	3.3
5_Member	GO Molecular Functions	GO:0019901	protein kinase binding	−6.8148	−3.13	#####	ADD2,RHOC,CCNT2,CDC25B,CDH1,CDH5,CRKL,CSPG4,DCX,DSP,FNTA,NFATC1,PAX6,PIK3R1,PIK3R2,PTK2,PTPRC,PTPRJ,SMO,STAT3,TOP2A,TRAF3,USF1,UTRN,MAP3K12,FZD5,NR4A3,SOCS1,KAT2B,CDK5R2,MAP3K13,TAOK2,BAG5,CIR1,TCL1B,RGS14,CLASP2,NBEAL2,STAU2,TEX14,SLC12A5,TBC1D14,ZBTB4,PITPNM3,TIRAP	45/699	45	6
5_Member	GO Molecular Functions	GO:0019900	kinase binding	−5.9174	−2.63	#####	ADD2,RHOC,CCNT2,CDC25B,CDH1,CDH5,CRKL,CSPG4,DCX,DSP,FNTA,NFATC1,PAX6,PIK3R1,PIK3R2,PTK2,PTPRC,PTPRJ,SMO,STAT3,TNFAIP3,TOP2A,TRAF3,USF1,UTRN,MAP3K12,FZD5,NR4A3,SOCS1,KAT2B,CDK5R2,MAP3K13,TAOK2,BAG5,CIR1,TCL1B,RGS14,CLASP2,NBEAL2,STAU2,TEX14,SLC12A5,TBC1D14,ZBTB4,PITPNM3,TIRAP	46/778	46	5.5
6_Member	GO Biological Processes	GO:0051129	negative regulation of cellular component organization	−6.6862	−3.07	#####	ADD2,APOD,B2M,CBLN1,CDH1,CDH5,DMRT1,EP300,GFI1,HSPA5,LGALS3,LMNA,MAP1B,MMP14,PIK3R1,PRTN3,PTGER4,RAD1,SVIL,TBCD,TOP2A,DYSF,KAT2B,USP10,BAG5,RUBCN,KNTC1,SPRY3,SEMA6C,ARFGEF1,KDM1A,CLASP2,PHF8,SYT11,CAMSAP2,CKAP2,CRBN,RTEL1,TEX14,SCAF4,SPTBN4,SEMA6D,ZDHHC12,NAV3,FRMD7,PRAG1	46/730	46	5.9
8_Member	GO Molecular Functions	GO:0008134	transcription factor binding	−6.2343	−2.73	#####	CCNT2,CRKL,E2F1,ELK1,EP300,FOS,GBX2,HOXA7,MEF2D,NFATC1,PAX6,PIM1,PPARD,RARB,RBBP8,RFC1,RXRA,SRY,STAT3,STAT5B,TAL1,TCF4,TWIST1,USF1,ZBTB16,NR4A3,KAT2B,PER2,RPL23,MED17,MED13,WWP2,PSIP1,BAZ2A,DDX20,SPEN,KDM1A,UBXN7,LCOR	39/592	39	5.8
8_Member	GO Cellular Components	GO:0005667	transcription regulator complex	−5.4261	−2.36	#####	BCL9,CHD3,E2F1,EP300,FOXE3,FOS,GFI1,JUNB,NFATC1,RBBP8,RXRA,SFPQ,STAT3,STAT5B,TAL1,TCF4,USF1,ZBTB16,NR4A3,TRRAP,PER2,LDB2,MED17,MAFB,IRF9,HAX1,HOXB13,DDX20,SPEN,KDM1A,ATF7IP,RCOR3,ALX4,LIN54	34/521	34	5.3
8_Member	GO Molecular Functions	GO:0140297	DNA-binding transcription factor binding	−5.1862	−2.24	#####	CRKL,E2F1,ELK1,EP300,FOS,GBX2,HOXA7,MEF2D,NFATC1,PPARD,RARB,RBBP8,RFC1,RXRA,SRY,STAT3,STAT5B,TAL1,TWIST1,USF1,NR4A3,KAT2B,MED17,MED13,WWP2,PSIP1,BAZ2A,DDX20,SPEN,KDM1A,UBXN7,LCOR	32/488	32	5.2
8_Member	GO Molecular Functions	GO:0061629	RNA polymerase II-specific DNA-binding transcription factor binding	−3.9345	−1.48	#####	CRKL,ELK1,EP300,FOS,GBX2,MEF2D,NFATC1,PPARD,RARB,RBBP8,RXRA,STAT3,STAT5B,TAL1,NR4A3,MED17,MED13,WWP2,BAZ2A,SPEN,KDM1A,UBXN7,LCOR	23/349	23	4.4
8_Member	GO Molecular Functions	GO:0016922	nuclear receptor binding	−2.3282	−0.59	#####	EP300,RARB,RXRA,STAT5B,NR4A3,MED17,MED13,BAZ2A,KDM1A,LCOR	10/137	10	3.3
9_Member	GO Biological Processes	GO:0060396	growth hormone receptor signaling pathway	−3.6588	−1.3	#####	CSH1,PIK3R1,PTK2,STAT3,STAT5B	#######	5	5.9
9_Member	GO Biological Processes	GO:0071378	cellular response to growth hormone stimulus	−3.5567	−1.25	#####	CSH1,PIK3R1,PTK2,STAT3,STAT5B	#######	5	5.7
9_Member	GO Cellular Components	GO:0005943	phosphatidylinositol 3-kinase complex, class IA	−2.8129	−0.85	#####	PIK3CG,PIK3R1,PIK3R2	#######	3	5.6
9_Member	GO Cellular Components	GO:0097651	phosphatidylinositol 3-kinase complex, class I	−2.8129	−0.85	#####	PIK3CG,PIK3R1,PIK3R2	#######	3	5.6
9_Member	GO Biological Processes	GO:0060416	response to growth hormone	−2.6511	−0.77	#####	CSH1,PIK3R1,PTK2,STAT3,STAT5B	5/34	5	4.3
9_Member	GO Biological Processes	GO:0043491	phosphatidylinositol 3-kinase/protein kinase B signal transduction	−2.5088	−0.69	#####	PIK3C2A,PIK3CG,PIK3R1,PIK3R2,PPARD,STAT3,PLEKHA1	7/70	7	3.7
9_Member	GO Cellular Components	GO:0005942	phosphatidylinositol 3-kinase complex	−2.0513	−0.45	#####	PIK3CG,PIK3R1,PIK3R2	#######	3	3.9
10_Member	GO Biological Processes	GO:0001568	blood vessel development	−6.0347	−2.65	#####	APOD,CASP8,CDH5,COL5A1,CRKL,CSPG4,CYP1B1,MEGF8,EGR3,EPHB4,VEGFD,FOLR1,GBX2,HOXA7,ID1,JUNB,MMP14,MYH9,MYLK,PAX6,PIK3CG,PTK2,PTPRJ,SMO,TAL1,PXDN,FZD5,CALCRL,HOXB13,ADAMTS6,SRPX2,SETD2,NSDHL,ZNF304,COL18A1,NRARP	36/534	36	5.7
10_Member	GO Biological Processes	GO:0001944	vasculature development	−5.6307	−2.42	#####	APOD,CASP8,CDH5,COL5A1,CRKL,CSPG4,CYP1B1,MEGF8,EGR3,EPHB4,VEGFD,FOLR1,GBX2,HOXA7,ID1,JUNB,MMP14,MYH9,MYLK,PAX6,PIK3CG,PTK2,PTPRJ,SMO,TAL1,PXDN,FZD5,CALCRL,HOXB13,ADAMTS6,SRPX2,SETD2,NSDHL,ZNF304,COL18A1,NRARP	36/556	36	5.4
10_Member	GO Biological Processes	GO:0048514	blood vessel morphogenesis	−5.2103	−2.24	#####	APOD,CASP8,CSPG4,CYP1B1,MEGF8,EGR3,EPHB4,VEGFD,FOLR1,GBX2,HOXA7,ID1,JUNB,MMP14,MYH9,MYLK,PIK3CG,PTK2,PTPRJ,SMO,TAL1,PXDN,FZD5,CALCRL,HOXB13,SRPX2,SETD2,ZNF304,COL18A1,NRARP	30/441	30	5.2
10_Member	GO Biological Processes	GO:0001525	angiogenesis	−4.862	−2.08	#####	APOD,CASP8,CSPG4,CYP1B1,EGR3,EPHB4,VEGFD,GBX2,HOXA7,ID1,MMP14,MYH9,PIK3CG,PTK2,PTPRJ,TAL1,PXDN,FZD5,CALCRL,HOXB13,SRPX2,SETD2,ZNF304,COL18A1,NRARP	25/347	25	5.1
10_Member	GO Biological Processes	GO:0035239	tube morphogenesis	−4.2685	−1.63	#####	APOD,CASP8,CSPG4,CYP1B1,MEGF8,EGR3,EPHB4,VEGFD,FOLR1,GBX2,HOXA7,HOXD11,HOXD13,ID1,JUNB,LAMA5,MMP14,MYH9,MYLK,PIK3CG,PTK2,PTPRJ,SMO,TAL1,TWIST1,PXDN,FZD5,CALCRL,HOXB13,SRPX2,CECR2,SETD2,ASB2,ZNF304,WNK4,COL18A1,CCDC39,NRARP	38/694	38	4.4
10_Member	GO Biological Processes	GO:0048562	embryonic organ morphogenesis	−3.1457	−1.03	#####	MEGF8,FOLR1,GBX2,HOXA7,HOXD11,MMP14,PAX6,RARB,SMO,TWIST1,ZIC1,FZD5,MAFB,TSHZ1,SETD2,ASB2,ATP8A2,ALX4,CCDC39	19/300	19	3.8
10_Member	GO Biological Processes	GO:0048598	embryonic morphogenesis	−2.8991	−0.9	#####	COL7A1,COL12A1,MEGF8,FOLR1,GBX2,HOXA7,HOXD11,HOXD13,LAMA5,MMP14,MMP15,MYH9,PAX6,RARB,SMO,TAL1,TWIST1,ZIC1,ZBTB16,FZD5,NR4A3,MAFB,TSHZ1,CECR2,SETD2,ASB2,ATP8A2,ALX4,TRIM15,CCDC39	30/597	30	3.4
10_Member	GO Biological Processes	GO:0048568	embryonic organ development	−2.7136	−0.8	#####	A2M,CASP8,MEGF8,FOLR1,GBX2,HOXA7,HOXD11,JUNB,MMP14,PAX6,RARB,SMO,TAL1,TWIST1,ZIC1,FZD5,MAFB,TSHZ1,SETD2,NSDHL,ASB2,ATP8A2,ALX4,CCDC39	24/454	24	3.3
10_Member	GO Biological Processes	GO:0007389	pattern specification process	−2.6277	−0.76	#####	C3,CRKL,MEGF8,EP300,FOLR1,GBX2,HOXA7,HOXD11,HOXD13,LAMA5,PAX6,SMO,STC1,ZIC1,ZBTB16,FZD5,MAFB,TSHZ1,ASB2,ALX4,PLD6,ANKS6,CCDC39,NRARP	24/461	24	3.2
10_Member	GO Biological Processes	GO:0003002	regionalization	−2.5749	−0.72	#####	C3,CRKL,MEGF8,EP300,FOLR1,GBX2,HOXA7,HOXD11,HOXD13,LAMA5,PAX6,SMO,ZBTB16,FZD5,MAFB,TSHZ1,ASB2,ALX4,PLD6,ANKS6,CCDC39,NRARP	22/413	22	3.2
10_Member	GO Biological Processes	GO:0043009	chordate embryonic development	−2.5467	−0.71	#####	ADCY9,CASP8,MEGF8,EP300,FOLR1,GBX2,HOXA7,HOXD11,JUNB,MMP14,MYH9,PAX6,RBBP8,SMO,TWIST1,FZD5,CIR1,POLG2,CECR2,SETD2,NSDHL,SBDS,KIAA1217,CHD8,ALX4,ZNF335,UPF3A,CMIP,UBR3,ANKS6,NRARP	31/658	31	3.1
10_Member	GO Biological Processes	GO:0048729	tissue morphogenesis	−2.5093	−0.69	#####	ADARB1,RHOC,COL5A1,DSP,MEGF8,FOLR1,GBX2,HOXD11,HOXD13,LAMA5,MMP14,MYLK,MYO9A,SMO,STC1,TAL1,TWIST1,FZD5,HOXB13,CECR2,SETD2,ASB2,WNK4,NKD1,TRIM15,KRT71,CCDC39,NRARP	28/579	28	3.1
10_Member	GO Biological Processes	GO:0009792	embryo development ending in birth or egg hatching	−2.3394	−0.59	#####	ADCY9,CASP8,MEGF8,EP300,FOLR1,GBX2,HOXA7,HOXD11,JUNB,MMP14,MYH9,PAX6,RBBP8,SMO,TWIST1,FZD5,CIR1,POLG2,CECR2,SETD2,NSDHL,SBDS,KIAA1217,CHD8,ALX4,ZNF335,UPF3A,CMIP,UBR3,ANKS6,NRARP	31/680	31	2.9
10_Member	GO Biological Processes	GO:0009952	anterior/posterior pattern specification	−2.1974	−0.52	#####	CRKL,EP300,GBX2,HOXA7,HOXD13,PAX6,SMO,ZBTB16,FZD5,TSHZ1,ALX4,PLD6,NRARP	13/213	13	3
10_Member	GO Biological Processes	GO:0002009	morphogenesis of an epithelium	−2.0172	−0.44	#####	RHOC,COL5A1,MEGF8,FOLR1,GBX2,HOXD11,HOXD13,LAMA5,MMP14,MYO9A,SMO,TWIST1,FZD5,HOXB13,CECR2,SETD2,ASB2,WNK4,NKD1,KRT71,CCDC39,NRARP	22/462	22	2.7
11_Member	GO Cellular Components	GO:0005813	centrosome	−5.92	−2.63	#####	ACADS,CDC25B,CHD3,E2F1,EYA3,IL4R,PTK2,TBCD,TCEA2,DYSF,DYRK3,GPAA1,KAT2B,ATP6V0D1,CIR1,CKAP5,PDCD6IP,RGS14,ENTR1,RAP1GAP2,CLASP2,EXOC7,CAMSAP2,NUP62,CKAP2,VPS4A,CFAP263,SPOUT1,HAUS6,CC2D1A,PXK,TTC12,KMT2E,KIAA1217,TGIF2,CCDC81,CCDC15,CEP78,CCDC77,NEK7,RILPL2,SKA3,RILPL1,RASSF10	44/729	44	5.5
11_Member	GO Biological Processes	GO:0051301	cell division	−3.1654	−1.04	#####	RHOC,CCNT2,CDC25B,MYH9,RBBP8,TOP2A,ZBTB16,DYRK3,KNTC1,CKAP5,PDCD6IP,RGS14,ENTR1,CLASP2,SMC5,EXOC7,CKAP2,VPS4A,CECR2,SPOUT1,EXOC6,HAUS6,TEX14,MICAL3,ANAPC1,MYH14,SENP5,SKA3	28/522	28	3.7
11_Member	GO Cellular Components	GO:0000922	spindle pole	−2.3658	−0.6	#####	CDC25B,DYNC1I1,KNTC1,CKAP5,RGS14,NUP62,CKAP2,VPS4A,SBDS,SPOUT1,NEK7,RASSF10	12/180	12	3.2
11_Member	GO Biological Processes	GO:0000278	mitotic cell cycle	−2.2709	−0.56	#####	RHOC,CDC25B,PAX6,RBBP8,STAT5B,TBCD,CLTCL1,TAOK2,KNTC1,CKAP5,PDCD6IP,RGS14,CLASP2,PHF8,SMC5,EXOC7,NUP62,CKAP2,VPS4A,SETD2,SBDS,GTSE1,RTEL1,EXOC6,TEX14,ANAPC1,MYH14,SKA3	28/603	28	2.9
11_Member	GO Cellular Components	GO:0005819	spindle	−2.0119	−0.43	#####	CDC25B,DYNC1I1,MYH9,CLTCL1,KAT2B,MAD2L1BP,KNTC1,CKAP5,RGS14,CLASP2,NUP62,CKAP2,VPS4A,SBDS,SPOUT1,HAUS6,MICAL3,CCAR2,NEK7,SKA3,RASSF10	21/435	21	2.7
13_Member	GO Biological Processes	GO:0030335	positive regulation of cell migration	−5.6807	−2.45	#####	ANXA3,RHOC,C5AR1,CASP8,CD74,CDH5,CRKL,CYP1B1,VEGFD,GCNT2,HSPA5,LBP,LGALS3,MMP14,MYLK,NTF3,NTRK3,PAX6,PIK3C2A,PIK3CG,PIK3R1,PTK2,PTPRC,PTPRJ,SMO,STAT3,TWIST1,NR4A3,TNFSF18,DNM1L,SEMA6C,CLASP2,SRPX2,ZNF304,DOCK5,SEMA6D,TIRAP,GLIPR2	38/600	38	5.4
13_Member	GO Biological Processes	GO:0040017	positive regulation of locomotion	−5.3187	−2.32	#####	ANXA3,RHOC,C5AR1,CASP8,CD74,CDH5,CRKL,CYP1B1,MEGF8,VEGFD,GCNT2,HSPA5,LBP,LGALS3,MMP14,MYLK,NTF3,NTRK3,PAX6,PIK3C2A,PIK3CG,PIK3R1,PTK2,PTPRC,PTPRJ,SMO,STAT3,TWIST1,NR4A3,TNFSF18,DNM1L,SEMA6C,CLASP2,SRPX2,ZNF304,DOCK5,SEMA6D,TIRAP,GLIPR2	39/646	39	5.2
13_Member	GO Biological Processes	GO:2000147	positive regulation of cell motility	−5.1892	−2.24	#####	ANXA3,RHOC,C5AR1,CASP8,CD74,CDH5,CRKL,CYP1B1,VEGFD,GCNT2,HSPA5,LBP,LGALS3,MMP14,MYLK,NTF3,NTRK3,PAX6,PIK3C2A,PIK3CG,PIK3R1,PTK2,PTPRC,PTPRJ,SMO,STAT3,TWIST1,NR4A3,TNFSF18,DNM1L,SEMA6C,CLASP2,SRPX2,ZNF304,DOCK5,SEMA6D,TIRAP,GLIPR2	38/630	38	5.1
13_Member	GO Biological Processes	GO:0071622	regulation of granulocyte chemotaxis	−3.9681	−1.48	#####	C5AR1,CD74,LBP,PTK2,PTPRJ,TNFSF18,DNM1L,TIRAP	8/54	8	5.4
13_Member	GO Biological Processes	GO:0002685	regulation of leukocyte migration	−3.5016	−1.22	#####	APOD,C5AR1,CD74,CRKL,VEGFD,HOXA7,LBP,LGALS3,MMP14,PIK3R1,PTGER4,PTK2,PTPRJ,ST3GAL4,TNFSF18,DNM1L,TIRAP	17/236	17	4.2
13_Member	GO Biological Processes	GO:0090023	positive regulation of neutrophil chemotaxis	−3.1987	−1.05	#####	C5AR1,CD74,LBP,DNM1L,TIRAP	#######	5	5.1
13_Member	GO Biological Processes	GO:0050921	positive regulation of chemotaxis	−3.131	−1.02	#####	C5AR1,CD74,MEGF8,VEGFD,LBP,NTF3,NTRK3,PTK2,PTPRJ,TNFSF18,DNM1L,TIRAP	12/146	12	4
13_Member	GO Biological Processes	GO:0071624	positive regulation of granulocyte chemotaxis	−3.044	−0.98	#####	C5AR1,CD74,LBP,DNM1L,TIRAP	#######	5	4.9
13_Member	GO Biological Processes	GO:1905523	positive regulation of macrophage migration	−3.044	−0.98	#####	C5AR1,MMP14,PTK2,PTPRJ,TNFSF18	#######	5	4.9
13_Member	GO Biological Processes	GO:0002687	positive regulation of leukocyte migration	−2.9775	−0.94	#####	C5AR1,CD74,VEGFD,LBP,LGALS3,MMP14,PIK3R1,PTK2,PTPRJ,TNFSF18,DNM1L,TIRAP	12/152	12	3.9
13_Member	GO Biological Processes	GO:0002690	positive regulation of leukocyte chemotaxis	−2.8773	−0.88	#####	C5AR1,CD74,VEGFD,LBP,PTK2,PTPRJ,TNFSF18,DNM1L,TIRAP	9/96	9	4
13_Member	GO Biological Processes	GO:0010759	positive regulation of macrophage chemotaxis	−2.7128	−0.8	#####	C5AR1,PTK2,PTPRJ,TNFSF18	#######	4	4.7
13_Member	GO Biological Processes	GO:0090022	regulation of neutrophil chemotaxis	−2.5939	−0.74	#####	C5AR1,CD74,LBP,DNM1L,TIRAP	5/35	5	4.2
13_Member	GO Biological Processes	GO:1902624	positive regulation of neutrophil migration	−2.5388	−0.71	#####	C5AR1,CD74,LBP,DNM1L,TIRAP	5/36	5	4.1
13_Member	GO Biological Processes	GO:0050920	regulation of chemotaxis	−2.1321	−0.49	#####	C5AR1,CD74,MEGF8,VEGFD,LBP,NTF3,NTRK3,PTK2,PTPRJ,TNFSF18,DNM1L,YTHDF1,TIRAP	13/217	13	2.9
13_Member	GO Biological Processes	GO:0010758	regulation of macrophage chemotaxis	−2.1073	−0.47	#####	C5AR1,PTK2,PTPRJ,TNFSF18	#######	4	3.6
13_Member	GO Biological Processes	GO:0002688	regulation of leukocyte chemotaxis	−2.0658	−0.45	#####	C5AR1,CD74,VEGFD,LBP,PTK2,PTPRJ,TNFSF18,DNM1L,TIRAP	9/127	9	3
13_Member	GO Biological Processes	GO:1905521	regulation of macrophage migration	−2.0366	−0.44	#####	C5AR1,MMP14,PTK2,PTPRJ,TNFSF18	5/47	5	3.3
14_Member	GO Molecular Functions	GO:0001216	DNA-binding transcription activator activity	−5.5556	−2.39	#####	CDC5L,DMRT1,E2F1,EGR3,ELK1,FOS,FOSB,GBX2,HOXA7,HOXD13,JUNB,MEF2D,NFATC1,NHLH1,PAX6,SHOX,SOX12,SRY,STAT3,STAT5B,TCF4,TFE3,USF1,ZIC1,ZBTB16,NR4A3,MAFB,PRDM4,ZNF319,ALX4,ZNF750,ZNF786,ARX	33/491	33	5.4
14_Member	GO Molecular Functions	GO:0001228	DNA-binding transcription activator activity, RNA polymerase II-specific	−5.2594	−2.27	#####	CDC5L,DMRT1,EGR3,ELK1,FOS,FOSB,GBX2,HOXA7,HOXD13,JUNB,MEF2D,NFATC1,NHLH1,PAX6,SHOX,SOX12,SRY,STAT3,STAT5B,TCF4,TFE3,USF1,ZIC1,ZBTB16,NR4A3,MAFB,PRDM4,ZNF319,ALX4,ZNF750,ZNF786,ARX	32/484	32	5.2
15_Member	GO Biological Processes	GO:1902105	regulation of leukocyte differentiation	−5.4951	−2.39	#####	CASP8,CD74,EGR3,EP300,FOS,HOXA7,IL4R,JUNB,MMP14,TNFRSF11B,PIK3R1,PTPRC,SLC4A2,SOX12,STAT5B,TAL1,TFE3,ZBTB16,SOCS1,TNFSF18,MAFB,HAX1,BRD7,LOXL3,EEIG1,NRARP	26/340	26	5.6
15_Member	GO Biological Processes	GO:1903706	regulation of hemopoiesis	−4.8737	−2.08	#####	B2M,CASP8,CD74,EGR3,EP300,FOS,HOXA7,IL4R,JUNB,MMP14,TNFRSF11B,PIK3R1,PTPRC,SLC4A2,SOX12,STAT3,STAT5B,TAL1,TFE3,ZBTB16,SOCS1,TNFSF18,MAFB,HAX1,KAT6B,BRD7,LOXL3,EEIG1,NRARP	29/436	29	5
15_Member	GO Biological Processes	GO:0007162	negative regulation of cell adhesion	−4.2149	−1.62	#####	APOD,CD74,CDH1,CYP1B1,GBP1,GCNT2,HOXA7,IL4R,LGALS3,MMP14,PIK3R1,PTK2,PTPRC,SLC4A2,TBCD,SOCS1,TNFSF18,SOCS6,CLASP2,TNFAIP8L2,LOXL3,NRARP	22/312	22	4.7
15_Member	GO Biological Processes	GO:0045580	regulation of T cell differentiation	−3.5294	−1.23	#####	CD74,EGR3,EP300,IL4R,JUNB,PTPRC,SLC4A2,SOX12,STAT5B,ZBTB16,SOCS1,TNFSF18,BRD7,LOXL3,NRARP	15/192	15	4.3
15_Member	GO Biological Processes	GO:1902107	positive regulation of leukocyte differentiation	−3.4574	−1.21	#####	CASP8,CD74,EGR3,EP300,FOS,IL4R,MMP14,PTPRC,SOX12,STAT5B,ZBTB16,SOCS1,HAX1,BRD7,EEIG1	15/195	15	4.2
15_Member	GO Biological Processes	GO:1903708	positive regulation of hemopoiesis	−3.4574	−1.21	#####	CASP8,CD74,EGR3,EP300,FOS,IL4R,MMP14,PTPRC,SOX12,STAT5B,ZBTB16,SOCS1,HAX1,BRD7,EEIG1	15/195	15	4.2
15_Member	GO Biological Processes	GO:1902106	negative regulation of leukocyte differentiation	−3.3114	−1.11	#####	CD74,HOXA7,IL4R,TNFRSF11B,PIK3R1,SLC4A2,SOCS1,TNFSF18,MAFB,LOXL3,NRARP	11/120	11	4.3
15_Member	GO Biological Processes	GO:0002683	negative regulation of immune system process	−3.2897	−1.1	#####	A2M,APOD,CD74,CD55,GBP1,HOXA7,IL4R,LGALS3,MNDA,OAS3,TNFRSF11B,PIM1,PIK3R1,PTGER4,PTPRC,PTPRJ,SLC4A2,TNFAIP3,TWIST1,SOCS1,TNFSF18,SOCS6,MAFB,SYT11,RNF115,TNFAIP8L2,LOXL3,ZDHHC12,NRARP,ACOD1	30/564	30	3.8
15_Member	GO Biological Processes	GO:0045619	regulation of lymphocyte differentiation	−3.2692	−1.08	#####	CD74,EGR3,EP300,IL4R,JUNB,MMP14,PTPRC,SLC4A2,SOX12,STAT5B,ZBTB16,SOCS1,TNFSF18,BRD7,LOXL3,NRARP	16/225	16	4
15_Member	GO Biological Processes	GO:0046634	regulation of alpha-beta T cell activation	−3.2205	−1.06	#####	CD55,EP300,IL4R,JUNB,LGALS3,PTPRC,SLC4A2,ZBTB16,SOCS1,TNFSF18,LOXL3	11/123	11	4.2
15_Member	GO Biological Processes	GO:0045581	negative regulation of T cell differentiation	−3.1752	−1.05	#####	CD74,IL4R,SLC4A2,SOCS1,TNFSF18,LOXL3,NRARP	7/54	7	4.6
15_Member	GO Biological Processes	GO:1903707	negative regulation of hemopoiesis	−3.1039	−1	#####	CD74,HOXA7,IL4R,TNFRSF11B,PIK3R1,SLC4A2,SOCS1,TNFSF18,MAFB,LOXL3,NRARP	11/127	11	4.1
15_Member	GO Biological Processes	GO:0046639	negative regulation of alpha-beta T cell differentiation	−2.9024	−0.9	#####	IL4R,SLC4A2,SOCS1,TNFSF18,LOXL3	#######	5	4.7
15_Member	GO Biological Processes	GO:0045621	positive regulation of lymphocyte differentiation	−2.8343	−0.85	#####	CD74,EGR3,EP300,IL4R,MMP14,PTPRC,SOX12,STAT5B,ZBTB16,SOCS1,BRD7	11/137	11	3.8
15_Member	GO Biological Processes	GO:0002695	negative regulation of leukocyte activation	−2.7862	−0.84	#####	CD74,IL4R,LGALS3,MNDA,PTPRC,SLC4A2,TNFAIP3,SOCS1,TNFSF18,SOCS6,SYT11,TNFAIP8L2,LOXL3,NRARP	14/204	14	3.6
15_Member	GO Biological Processes	GO:0046637	regulation of alpha-beta T cell differentiation	−2.7599	−0.82	#####	EP300,IL4R,JUNB,SLC4A2,ZBTB16,SOCS1,TNFSF18,LOXL3	8/81	8	3.9
15_Member	GO Biological Processes	GO:0045620	negative regulation of lymphocyte differentiation	−2.7329	−0.81	#####	CD74,IL4R,SLC4A2,SOCS1,TNFSF18,LOXL3,NRARP	7/64	7	4
15_Member	GO Biological Processes	GO:0045582	positive regulation of T cell differentiation	−2.6919	−0.79	#####	CD74,EGR3,EP300,IL4R,PTPRC,SOX12,STAT5B,ZBTB16,SOCS1,BRD7	10/122	10	3.7
15_Member	GO Biological Processes	GO:0010453	regulation of cell fate commitment	−2.6631	−0.78	#####	CHD3,EP300,PAX6,TNFSF18,ESRP1,LOXL3	6/49	6	4.1
15_Member	GO Biological Processes	GO:0022407	regulation of cell–cell adhesion	−2.6392	−0.76	#####	B2M,CD74,CDH1,CD55,EGR3,EP300,GCNT2,IL4R,LGALS3,PTK2,PTPRC,ST3GAL4,SLC4A2,SOX12,STAT5B,ZBTB16,NR4A3,SOCS1,TNFSF18,SOCS6,PKP3,BRD7,TNFAIP8L2,MEGF10,LOXL3,NRARP	26/513	26	3.2
15_Member	GO Biological Processes	GO:0046636	negative regulation of alpha-beta T cell activation	−2.6176	−0.75	#####	IL4R,LGALS3,SLC4A2,SOCS1,TNFSF18,LOXL3	6/50	6	4
15_Member	GO Biological Processes	GO:0010721	negative regulation of cell development	−2.6012	−0.74	#####	B2M,CD74,CDH1,HOXA7,IL4R,TNFRSF11B,PAX6,PIK3R1,SLC4A2,ABCC8,SOCS1,TNFSF18,MAFB,SEMA6C,SEMA6D,LOXL3,NRARP	17/285	17	3.3
15_Member	GO Biological Processes	GO:0002697	regulation of immune effector process	−2.5622	−0.72	#####	A2M,AP1G1,B2M,C3,CD1A,CD74,CD55,DNASE1L3,EP300,IL4R,JUNB,LAMP1,LBP,LGALS3,PTPRC,STAT5B,TWIST1,FZD5,NR4A3,TNFSF18,PKP3,LOXL3	22/414	22	3.2
15_Member	GO Biological Processes	GO:0051250	negative regulation of lymphocyte activation	−2.5044	−0.69	#####	CD74,IL4R,LGALS3,MNDA,SLC4A2,TNFAIP3,SOCS1,TNFSF18,SOCS6,TNFAIP8L2,LOXL3,NRARP	12/173	12	3.4
15_Member	GO Biological Processes	GO:1903037	regulation of leukocyte cell–cell adhesion	−2.4737	−0.67	#####	B2M,CD74,CD55,EGR3,EP300,IL4R,LGALS3,PTPRC,ST3GAL4,SLC4A2,SOX12,STAT5B,ZBTB16,NR4A3,SOCS1,TNFSF18,SOCS6,BRD7,TNFAIP8L2,LOXL3,NRARP	21/395	21	3.1
15_Member	GO Biological Processes	GO:0002694	regulation of leukocyte activation	−2.3677	−0.6	#####	B2M,CD74,CD55,EGR3,EP300,IL4R,JUNB,LBP,LGALS3,MMP14,MNDA,PTPRC,SLC4A2,SOX12,STAT5B,TNFAIP3,ZBTB16,NR4A3,SOCS1,TNFSF18,SOCS6,SYT11,BRD7,ZNF335,TNFAIP8L2,LOXL3,TIRAP,NRARP	28/593	28	3
15_Member	GO Biological Processes	GO:0050866	negative regulation of cell activation	−2.3649	−0.6	#####	CD74,IL4R,LGALS3,MNDA,PTPRC,SLC4A2,TNFAIP3,SOCS1,TNFSF18,SOCS6,SYT11,TNFAIP8L2,LOXL3,NRARP	14/227	14	3.2
15_Member	GO Biological Processes	GO:0050868	negative regulation of T cell activation	−2.3506	−0.6	#####	CD74,IL4R,LGALS3,SLC4A2,SOCS1,TNFSF18,SOCS6,TNFAIP8L2,LOXL3,NRARP	10/136	10	3.3
15_Member	GO Biological Processes	GO:0051249	regulation of lymphocyte activation	−2.2588	−0.55	#####	B2M,CD74,CD55,EGR3,EP300,IL4R,JUNB,LGALS3,MMP14,MNDA,PTPRC,SLC4A2,SOX12,STAT5B,TNFAIP3,ZBTB16,SOCS1,TNFSF18,SOCS6,BRD7,ZNF335,TNFAIP8L2,LOXL3,TIRAP,NRARP	25/521	25	2.9
15_Member	GO Biological Processes	GO:0045785	positive regulation of cell adhesion	−2.2024	−0.52	#####	B2M,CD74,CRKL,CD55,EGR3,EP300,GCNT2,IL4R,PIK3R2,PTPRC,PTPRJ,ST3GAL4,SOX12,STAT5B,TFE3,UTRN,ZBTB16,NR4A3,SOCS1,TNFSF18,PKP3,BRD7,DOCK5,MEGF10	24/499	24	2.8
15_Member	GO Biological Processes	GO:0050863	regulation of T cell activation	−2.1544	−0.5	#####	B2M,CD74,CD55,EGR3,EP300,IL4R,JUNB,LGALS3,PTPRC,SLC4A2,SOX12,STAT5B,ZBTB16,SOCS1,TNFSF18,SOCS6,BRD7,TNFAIP8L2,LOXL3,NRARP	20/395	20	2.8
15_Member	GO Biological Processes	GO:2000328	regulation of T-helper 17 cell lineage commitment	−2.1326	−0.49	#####	EP300,TNFSF18,LOXL3	#######	3	4.1
15_Member	GO Biological Processes	GO:0043370	regulation of CD4-positive, alpha-beta T cell differentiation	−2.1168	−0.48	#####	EP300,IL4R,JUNB,SOCS1,TNFSF18,LOXL3	6/63	6	3.3
15_Member	GO Biological Processes	GO:2000319	regulation of T-helper 17 cell differentiation	−2.1073	−0.47	#####	EP300,JUNB,TNFSF18,LOXL3	#######	4	3.6
15_Member	GO Biological Processes	GO:0045596	negative regulation of cell differentiation	−2.0796	−0.45	#####	B2M,CD74,CDH1,COL5A1,E2F1,FOXE3,HOXA7,IL4R,NFATC1,TNFRSF11B,PAX6,PIK3R1,PPARD,RARB,SLC4A2,SMO,STAT3,STAT5B,ABCC8,TWIST1,ZBTB16,SOCS1,TNFSF18,MAFB,SEMA6C,MBNL3,BRD9,ZNF750,SEMA6D,ADGRV1,LOXL3,NRARP	32/739	32	2.7
15_Member	GO Biological Processes	GO:0050865	regulation of cell activation	−2.0748	−0.45	#####	B2M,CD74,CD55,EGR3,EP300,IL4R,JUNB,LBP,LGALS3,MMP14,MNDA,PTPRC,PTPRJ,SLC4A2,SOX12,STAT5B,TNFAIP3,ZBTB16,NR4A3,SOCS1,TNFSF18,SOCS6,SYT11,BRD7,ZNF335,TNFAIP8L2,LOXL3,TIRAP,NRARP	29/653	29	2.7
15_Member	GO Biological Processes	GO:2000514	regulation of CD4-positive, alpha-beta T cell activation	−2.0468	−0.45	#####	CD55,EP300,IL4R,JUNB,SOCS1,TNFSF18,LOXL3	7/85	7	3.1
16_Member	GO Biological Processes	GO:0061564	axon development	−5.4877	−2.39	#####	ADARB1,APOD,MEGF8,FOLR1,GBX2,MAP1B,NCAM1,NCAM2,PAX6,PTK2,PTPRJ,SCN1B,SMO,TOP2B,CDK5R2,KALRN,TAOK2,MYOT,SEMA6C,SLITRK3,NFASC,CAMSAP2,ATP8A2,SPTBN4,NEUROG2,SEMA6D,SLITRK2,PLEKHG4B,ARX,OR10A4	30/427	30	5.4
16_Member	GO Biological Processes	GO:0031175	neuron projection development	−4.9931	−2.15	#####	ADARB1,APOD,CDH1,CRKL,MEGF8,FOLR1,GBX2,MAP1B,NCAM1,NCAM2,NTF3,PAX6,PTK2,PTPRJ,SCN1B,SMO,TOP2B,CDK5R2,KALRN,TAOK2,MYOT,SEMA6C,SLITRK3,NFASC,CAMSAP2,CECR2,ATP8A2,CC2D1A,UBA6,DOCK10,SLC12A5,SPTBN4,ZNF335,NEUROG2,SEMA6D,ADGRV1,SLITRK2,PLEKHG4B,ARX,ADGRF1,OR10A4	41/717	41	4.9
16_Member	GO Biological Processes	GO:0000902	cell morphogenesis	−4.7944	−2.02	#####	ADARB1,CDH1,CDH5,DMRT1,EP300,GBX2,MAP1B,MYH9,NCAM1,NTF3,PAX6,PTK2,PTPRJ,SCN1B,SMO,STC1,TAL1,TBCD,TOP2B,CDK5R2,KALRN,TAOK2,MYOT,SEMA6C,SLITRK3,NFASC,NBEAL2,ATP8A2,CC2D1A,DOCK10,SPTBN4,ZNF335,SEMA6D,COL18A1,SLITRK2,PLEKHG4B,ARX,RILPL2,OR10A4,RILPL1	40/706	40	4.8
16_Member	GO Biological Processes	GO:0007409	axonogenesis	−3.9731	−1.48	#####	ADARB1,GBX2,MAP1B,NCAM1,PAX6,PTK2,PTPRJ,SCN1B,SMO,TOP2B,CDK5R2,KALRN,TAOK2,MYOT,SEMA6C,SLITRK3,NFASC,ATP8A2,SPTBN4,SEMA6D,SLITRK2,PLEKHG4B,ARX,OR10A4	24/370	24	4.4
16_Member	GO Biological Processes	GO:0048667	cell morphogenesis involved in neuron differentiation	−3.8436	−1.42	#####	ADARB1,GBX2,MAP1B,NCAM1,PAX6,PTK2,PTPRJ,SCN1B,SMO,TBCD,TOP2B,CDK5R2,KALRN,TAOK2,MYOT,SEMA6C,SLITRK3,NFASC,ATP8A2,CC2D1A,DOCK10,SPTBN4,SEMA6D,SLITRK2,PLEKHG4B,ARX,OR10A4	27/449	27	4.3
16_Member	GO Biological Processes	GO:0048812	neuron projection morphogenesis	−3.4849	−1.21	#####	ADARB1,GBX2,MAP1B,NCAM1,NTF3,PAX6,PTK2,PTPRJ,SCN1B,SMO,TOP2B,CDK5R2,KALRN,TAOK2,MYOT,SEMA6C,SLITRK3,NFASC,ATP8A2,CC2D1A,DOCK10,SPTBN4,ZNF335,SEMA6D,SLITRK2,PLEKHG4B,ARX,OR10A4	28/498	28	4
16_Member	GO Biological Processes	GO:0120039	plasma membrane bounded cell projection morphogenesis	−3.416	−1.17	#####	ADARB1,GBX2,MAP1B,NCAM1,NTF3,PAX6,PTK2,PTPRJ,SCN1B,SMO,TOP2B,CDK5R2,KALRN,TAOK2,MYOT,SEMA6C,SLITRK3,NFASC,ATP8A2,CC2D1A,DOCK10,SPTBN4,ZNF335,SEMA6D,SLITRK2,PLEKHG4B,ARX,OR10A4	28/503	28	3.9
16_Member	GO Biological Processes	GO:0048858	cell projection morphogenesis	−3.3216	−1.12	#####	ADARB1,GBX2,MAP1B,NCAM1,NTF3,PAX6,PTK2,PTPRJ,SCN1B,SMO,TOP2B,CDK5R2,KALRN,TAOK2,MYOT,SEMA6C,SLITRK3,NFASC,ATP8A2,CC2D1A,DOCK10,SPTBN4,ZNF335,SEMA6D,SLITRK2,PLEKHG4B,ARX,OR10A4	28/510	28	3.8
16_Member	GO Biological Processes	GO:0007411	axon guidance	−3.2068	−1.05	#####	GBX2,NCAM1,PAX6,PTK2,PTPRJ,SCN1B,SMO,CDK5R2,KALRN,MYOT,SEMA6C,NFASC,SEMA6D,PLEKHG4B,ARX,OR10A4	16/228	16	4
16_Member	GO Biological Processes	GO:0097485	neuron projection guidance	−3.1863	−1.05	#####	GBX2,NCAM1,PAX6,PTK2,PTPRJ,SCN1B,SMO,CDK5R2,KALRN,MYOT,SEMA6C,NFASC,SEMA6D,PLEKHG4B,ARX,OR10A4	16/229	16	3.9
17_Member	GO Biological Processes	GO:0071345	cellular response to cytokine stimulus	−3.4736	−1.21	#####	CD74,CNTFR,CRKL,CYP1B1,EPHB4,FOS,GBP1,HSPA5,IFNA10,IFNA14,IFNGR1,IL4R,MNDA,NFATC1,NTRK3,OAS3,PAX6,PIM1,PIK3R1,PRL,PTPRJ,ABCD4,ROS1,CXCL11,STAT3,STAT5B,TRAF3,KMO,SOCS1,TNFSF18,USP10,HAX1,CCR9,TEX14,IFNK,EPG5,EDA2R,MYLK3,ACOD1	39/787	39	3.8
17_Member	GO Biological Processes	GO:0019221	cytokine-mediated signaling pathway	−3.0146	−0.96	#####	CD74,CNTFR,EPHB4,IFNA10,IFNA14,IFNGR1,IL4R,NTRK3,OAS3,PIK3R1,PRL,PTPRJ,ROS1,CXCL11,STAT3,STAT5B,TRAF3,SOCS1,TNFSF18,HAX1,CCR9,IFNK,EPG5,EDA2R	24/431	24	3.6
18_Member	GO Biological Processes	GO:0010038	response to metal ion	−5.3573	−2.34	#####	AMELX,B2M,CASP8,CDH1,DPEP1,FOS,FOSB,JUNB,KCNC1,TNFRSF11B,OTC,ABCC8,SLC30A3,CACNA1G,LONP1,TESMIN,AHCYL1,SETD2,EEF2K,SLC25A39,ADGRV1,MAP1LC3A,CYP2R1,NEK7,MICU2,CPNE2	26/346	26	5.4
18_Member	GO Biological Processes	GO:0071277	cellular response to calcium ion	−2.866	−0.87	#####	DPEP1,FOS,FOSB,JUNB,EEF2K,ADGRV1,MICU2,CPNE2	8/78	8	4.1
18_Member	GO Biological Processes	GO:0071248	cellular response to metal ion	−2.7803	−0.83	#####	B2M,CDH1,DPEP1,FOS,FOSB,JUNB,EEF2K,SLC25A39,ADGRV1,MAP1LC3A,NEK7,MICU2,CPNE2	13/182	13	3.6
18_Member	GO Biological Processes	GO:0051592	response to calcium ion	−2.3061	−0.58	#####	AMELX,DPEP1,FOS,FOSB,JUNB,AHCYL1,EEF2K,ADGRV1,MICU2,CPNE2	10/138	10	3.2
19_Member	GO Molecular Functions	GO:0140657	ATP-dependent activity	−5.1541	−2.23	#####	ABCD1,CHD2,CHD3,DYNC1I1,HSPA5,MYH9,MYO9A,PEX6,ABCD4,RFC1,ABCC8,TOP2A,TOP2B,ATP6V0D1,LONP1,DDX20,ATP10B,SMC5,MDN1,SMCHD1,ABCA6,VPS4A,TOR2A,CECR2,RTEL1,ATP8A2,DDX56,TOR4A,UBA6,ATF7IP,TMEM30A,CHD8,MYH14,FBH1,NAV3,SLFN11	36/584	36	5.1
19_Member	GO Molecular Functions	GO:0016887	ATP hydrolysis activity	−3.9731	−1.48	#####	ABCD1,CHD2,CHD3,HSPA5,PEX6,ABCD4,RFC1,ABCC8,LONP1,DDX20,ATP10B,SMC5,MDN1,SMCHD1,ABCA6,VPS4A,TOR2A,RTEL1,ATP8A2,DDX56,TOR4A,ATF7IP,CHD8,FBH1,NAV3,SLFN11	26/417	26	4.4
19_Member	GO Molecular Functions	GO:0140097	catalytic activity, acting on DNA	−3.3559	−1.14	#####	CHD2,CHD3,DNASE1L3,HSPA5,RAD1,RBBP8,RFC1,TOP2A,TOP2B,DDX20,POLG2,SMCHD1,VPS4A,POLM,CECR2,RTEL1,DDX56,CHD8,FTO,FBH1,POLN	21/335	21	4
19_Member	GO Molecular Functions	GO:0140640	catalytic activity, acting on a nucleic acid	−3.0291	−0.97	#####	CHD2,CHD3,DNASE1L3,HSPA5,RAD1,RBBP8,RFC1,TOP2A,TOP2B,N4BP1,POP1,DDX20,POLG2,SMCHD1,SND1,VPS4A,POLM,CECR2,RTEL1,DDX56,CDKAL1,NYNRIN,CHD8,FTO,USB1,FBH1,SLFN11,MTFMT,PIWIL4,PLD6,ZC3H12B,POLN	32/639	32	3.5
19_Member	GO Molecular Functions	GO:0017111	ribonucleoside triphosphate phosphatase activity	−2.8203	−0.85	#####	ABCD1,RHOC,CHD2,CHD3,GBP1,ERAS,HSPA5,PEX6,ABCD4,RFC1,ABCC8,LONP1,DNM1L,RGS14,DDX20,ATP10B,SMC5,MDN1,SMCHD1,ABCA6,VPS4A,TOR2A,RTEL1,ATP8A2,DDX56,TOR4A,LSG1,ATF7IP,CHD8,FBH1,NAV3,SLFN11,AGAP4,DIRAS1,AGAP6	35/741	35	3.3
19_Member	GO Molecular Functions	GO:0008094	ATP-dependent activity, acting on DNA	−2.7076	−0.79	#####	CHD2,CHD3,HSPA5,RFC1,TOP2A,TOP2B,DDX20,SMCHD1,VPS4A,CECR2,RTEL1,DDX56,CHD8,FBH1	14/208	14	3.5
19_Member	GO Molecular Functions	GO:0140658	ATP-dependent chromatin remodeler activity	−2.3522	−0.6	#####	CHD2,CHD3,HSPA5,DDX20,SMCHD1,VPS4A,CECR2,RTEL1,DDX56,CHD8,FBH1	11/158	11	3.2
19_Member	GO Molecular Functions	GO:0003689	DNA clamp loader activity	−2.3113	−0.58	#####	CHD2,CHD3,HSPA5,RFC1,DDX20,SMCHD1,VPS4A,RTEL1,DDX56,CHD8,FBH1	11/160	11	3.2
19_Member	GO Biological Processes	GO:0140588	chromatin looping	−2.1744	−0.51	#####	CHD2,CHD3,HSPA5,DDX20,SMC5,SMCHD1,VPS4A,RTEL1,DDX56,CHD8,FBH1	11/167	11	3
19_Member	GO Biological Processes	GO:0071103	DNA conformation change	−2.0701	−0.45	#####	CHD2,CHD3,RPA1,TOP2A,TOP2B,RTEL1,CHD8,FBH1	8/105	8	3.1
19_Member	GO Molecular Functions	GO:0000510	H3-H4 histone complex chaperone activity	−2.039	−0.44	#####	CHD2,CHD3,HSPA5,DDX20,SMCHD1,VPS4A,RTEL1,DDX56,CHD8,FBH1	10/151	10	2.9
19_Member	GO Molecular Functions	GO:0140584	chromatin extrusion motor activity	−2.039	−0.44	#####	CHD2,CHD3,HSPA5,DDX20,SMCHD1,VPS4A,RTEL1,DDX56,CHD8,FBH1	10/151	10	2.9
19_Member	GO Molecular Functions	GO:0140665	ATP-dependent H3-H4 histone complex chaperone activity	−2.039	−0.44	#####	CHD2,CHD3,HSPA5,DDX20,SMCHD1,VPS4A,RTEL1,DDX56,CHD8,FBH1	10/151	10	2.9
19_Member	GO Molecular Functions	GO:0140849	ATP-dependent H2AZ histone chaperone activity	−2.039	−0.44	#####	CHD2,CHD3,HSPA5,DDX20,SMCHD1,VPS4A,RTEL1,DDX56,CHD8,FBH1	10/151	10	2.9
19_Member	GO Molecular Functions	GO:0061749	forked DNA-dependent helicase activity	−2.0366	−0.44	#####	CHD2,CHD3,RTEL1,CHD8,FBH1	5/47	5	3.3
19_Member	GO Molecular Functions	GO:1990518	single-stranded 3′-5' DNA helicase activity	−2.0366	−0.44	#####	CHD2,CHD3,RTEL1,CHD8,FBH1	5/47	5	3.3
19_Member	GO Molecular Functions	GO:0015616	DNA translocase activity	−2.0199	−0.44	#####	CHD2,CHD3,HSPA5,DDX20,SMCHD1,VPS4A,RTEL1,DDX56,CHD8,FBH1	10/152	10	2.9
19_Member	GO Molecular Functions	GO:0140674	ATP-dependent histone chaperone activity	−2.0199	−0.44	#####	CHD2,CHD3,HSPA5,DDX20,SMCHD1,VPS4A,RTEL1,DDX56,CHD8,FBH1	10/152	10	2.9
19_Member	GO Molecular Functions	GO:0061775	cohesin loader activity	−2.001	−0.43	#####	CHD2,CHD3,HSPA5,DDX20,SMCHD1,VPS4A,RTEL1,DDX56,CHD8,FBH1	10/153	10	2.9

To further characterize the molecular mechanisms distinguishing cancer types, we performed KEGG pathway enrichment analysis on the top 1000 classification-driving genes (Table 4). Figure 6B illustrates significant enrichment of multiple cancer-relevant pathways. JAK–STAT signaling (hsa04630, *P* = 4.28 × 10^−6^) emerged as the most significantly enriched pathway with 17 regulated genes, underscoring its central role in cancer type-specific signaling. Several pathways related to viral carcinogenesis were prominently enriched, including Epstein–Barr virus infection (hsa05169, *P* = 5.61 × 10^−4^), hepatitis B (hsa05161, *P* = 1.20 × 10^−5^), and human papillomavirus infection (hsa05165, *P* = 3.28 × 10^−5^), reflecting the established etiological role of viral infections in specific cancer types. Acute myeloid leukemia pathway (hsa05221, *P* = 9.96 × 10^−5^) showed the highest rich factor (0.132), suggesting particularly strong relevance despite involving fewer genes. TNF signaling (hsa04668, *P* = 4.53 × 10^−4^) and cytokine-cytokine receptor interaction (hsa04060, *P* = 8.83 × 10^−3^) pathways were significantly enriched, indicating important differences in inflammatory response across cancer types. Small cell lung cancer pathway (hsa05222, *P* = 4.12 × 10^−3^) and non-small cell lung cancer pathway (hsa05223, *P* = 3.92 × 10^−3^) enrichment corroborate the inclusion of lung cancer subtypes in our analysis. Prolactin signaling (hsa04917, *P* = 7.28 × 10^−4^) and ErbB signaling (hsa04012, *P* = 9.54 × 10^−3^) pathways, both involved in growth factor signaling, exhibited notable enrichment. These results highlight the distinct signaling networks that define and differentiate major cancer types.

**Table 4:** KEGG pathway enrichment analysis result.

**KEGGterm**	**Category**	**Description**	**total number**	**term number**	***P-*value**	**regulated**	**rich_factor**	rich_factor*6
hsa05221	KEGG Pathway	Acute myeloid leukemia	847	68	9.956579010296642e-05	9	0.13	0.792
hsa04935	KEGG Pathway	Growth hormone synthesis, secretion and action	847	122	1.413772993250137e-04	12	0.1	0.588
hsa04668	KEGG Pathway	TNF signaling pathway	847	119	4.531485865651061e-04	11	0.09	0.552
hsa05203	KEGG Pathway	Viral carcinogenesis	847	205	5.888436550840925e-04	15	0.07	0.438
hsa04917	KEGG Pathway	Prolactin signaling pathway	847	71	7.283551762929163e-04	8	0.11	0.678
hsa05223	KEGG Pathway	Non-small cell lung cancer	847	73	3.923338614721054e-03	7	0.1	0.576
hsa05222	KEGG Pathway	Small cell lung cancer	847	93	4.119737089772278e-03	8	0.09	0.516
hsa04062	KEGG Pathway	Chemokine signaling pathway	847	193	7.413126253150530e-03	12	0.06	0.372
hsa04012	KEGG Pathway	ErbB signaling pathway	847	86	9.543842827385311e-03	7	0.08	0.486
hsa04630	KEGG Pathway	JAK–STAT signaling pathway	847	168	4.282544466484046e-06	17	0.1	0.606
hsa04060	KEGG Pathway	Cytokine-cytokine receptor interaction	847	298	8.831763135891698e-03	16	0.05	0.324
hsa05161	KEGG Pathway	Hepatitis B	847	163	1.199279412037945e-05	16	0.1	0.588
hsa05167	KEGG Pathway	Kaposi sarcoma-associated herpesvirus infection	847	196	3.280684461878119e-05	17	0.09	0.522
hsa05162	KEGG Pathway	Measles	847	139	1.272111055330517e-04	13	0.09	0.564
hsa04620	KEGG Pathway	Toll-like receptor signaling pathway	847	109	2.113489987063978e-04	11	0.1	0.606
hsa05171	KEGG Pathway	Coronavirus disease - COVID-19	847	238	3.471081216715737e-04	17	0.07	0.426
hsa05152	KEGG Pathway	Tuberculosis	847	182	5.428785647420490e-04	14	0.08	0.462
hsa05169	KEGG Pathway	Epstein–Barr virus infection	847	204	5.614610525771224e-04	15	0.07	0.444
hsa04621	KEGG Pathway	NOD-like receptor signaling pathway	847	189	7.875531193662043e-04	14	0.07	0.444
hsa05160	KEGG Pathway	Hepatitis C	847	159	1.551451997432882e-03	12	0.08	0.45
hsa05417	KEGG Pathway	Lipid and atherosclerosis	847	216	2.764175030388518e-03	14	0.07	0.39
hsa05164	KEGG Pathway	Influenza A	847	173	3.119625071556026e-03	12	0.07	0.414
hsa04622	KEGG Pathway	RIG-I-like receptor signaling pathway	847	72	3.630165704656456e-03	7	0.1	0.582
hsa05168	KEGG Pathway	Herpes simplex virus 1 infection	847	182	4.698786512943331e-03	12	0.07	0.396
hsa04936	KEGG Pathway	Alcoholic liver disease	847	144	6.630779483506825e-03	10	0.07	0.414

### Comprehensive performance assessment of Conv1D versus traditional machine learning models

**Figure 7:**
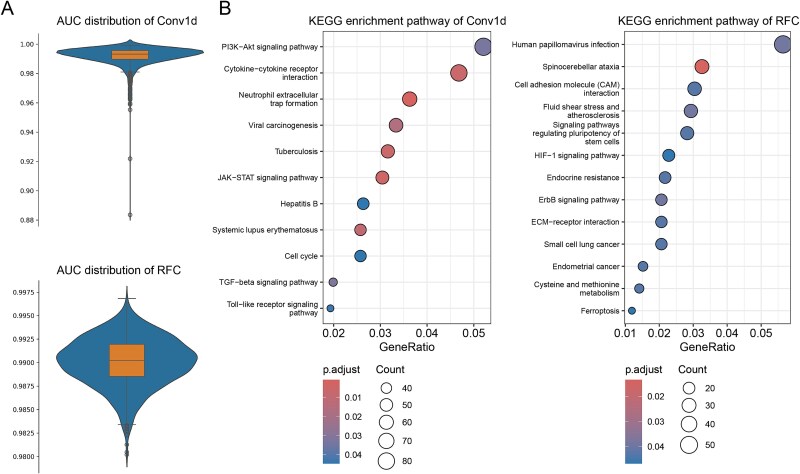
Performance comparison between Conv1D and RFC classification models. (A) Distribution of AUC values obtained from 1000 runs of the Conv1D and RFC models; (B) KEGG pathway enrichment analysis results based on significant features identified from 1000 runs of the Conv1D and RFC models.

To benchmark the Conv1D model against traditional machine learning approaches, we trained Random Forest (RFC), Logistic Regression (LR), and k-Nearest Neighbor (KNN) classifiers on the same 8-cancer classification task, performing 1000 independent runs for Conv1D and RFC. Both Conv1D and RFC demonstrated strong and stable discriminatory performance with near-ceiling AUC values (Fig. 7A). The Conv1D model achieved a mean AUC of 0.9916 (median = 0.9932; bootstrap 95% CI: [0.9911, 0.9920]) and a mean accuracy of 0.9296 (95% CI: [0.9283, 0.9310]). In comparison, RFC yielded a mean AUC of 0.9901 (median = 0.9902; 95% CI: [0.9899, 0.9902]) and mean accuracy of 0.9140 (95% CI: [0.9133, 0.9147]), while LR achieved an accuracy of 0.8871 and AUC of 0.9885, and KNN reached an accuracy of 0.8571 with AUC of 0.9740.

AUC values of Conv1D, RFC, and LR were all significantly greater than random performance (AUC = 0.5; one-sided Wilcoxon signed-rank test, *P* < .001). While the Conv1D model exhibited moderately higher performance variability across runs, it consistently achieved superior median and upper-quartile AUC values. This pattern suggests that Conv1D is more capable of capturing complex, non-linear mutational patterns when adequately trained, albeit at the cost of increased sensitivity to initialization and training conditions.

Functional enrichment analysis of significantly stable features (FDR < 0.05) revealed fundamental differences in biological interpretability between the models (Fig. 7B). The Conv1D model identified 3776 significantly stable genes—60% more than RFC's significantly stable 2353 genes—with enrichment of cancer-type-specific pathways directly relevant to the classification task (Table 5, Supplementary Tables S5 and S6). Top-ranked Conv1D pathways included ‘Neutrophil extracellular trap formation’ (KEGG hsa04613, 62 genes, q = 0.0006; promotes progression in HCC, breast, pancreatic, gastric, ovarian, colon cancers via invasion, metastasis, EMT [25, 26]), ‘JAK-STAT signaling pathway’ (hsa04630, 52 genes, q = 0.0039; drives proliferation, survival, invasion, angiogenesis, immune suppression across multiple cancers [27]), ‘Cytokine-cytokine receptor interaction’ (hsa04060, 80 genes, q = 0.0055; enhances survival, proliferation, chemoresistance in epithelial cancers [28]), and tissue-specific drivers such as "Hepatitis B″ for LIHC (hsa05161, 45 genes, q = 0.064; promotes HCC via chronic inflammation, oncogenesis [29]), "Tuberculosis" for lung cancers (hsa05152, 54 genes, q = 0.0055; chronic *Mycobacterium tuberculosis* infection promotes lung carcinogenesis through sustained inflammation and oxidative stress [30]), and immune processes like ‘negative regulation of interleukin-2 production’ for other immune microenvironments (GO:0032703, 15 genes, q = 0.035; contributes to immune evasion, suppression [31, 32]). These pathways mechanistically align with known cancer biology: chronic inflammation driving COAD/STAD/OV tumorigenesis via NF-κB/STAT3, cytokine secretion, viral oncogenesis in LIHC, and immune dysregulation in ovarian cancer via PD-L1, TAMs/Tregs [33–35]. In stark contrast, RFC top enriched pathways were dominated by neuronal and generic processes mechanistically irrelevant to epithelial carcinoma pathogenesis, such as "Spinocerebellar ataxia" (hsa05017, 30 genes, q = 0.012; neurodegenerative, no direct role in carcinomas) and "Dopaminergic synapse" (hsa04728, 23 genes, q = 0.065; limited, context-dependent in some tumors like GBM but irrelevant broadly), despite including some relevant terms like ‘Human papillomavirus infection’ for CESC (hsa05165, 52 genes, q = 0.034; promotes via transformation), likely reflecting dataset-specific noise.

**Table 5:** Enrichment analysis based on significant features identified from 1000 runs of the Conv1D and RFC models.

1,GO pathway enrichment analysis of the Conv1D										
ONTOLOGY		ID	Description	GeneRatio	BgRatio	pvalue	p.adjust	qvalue	geneID	Count
BP		GO:0032703	negative regulation of interleukin-2 production	15/3375	28/18870	2.10457927126818e-05	0.0367689447889173	0.0350009391068565	7430/5621/84868/50943/9455/7128/3902/10524/64127/5987/51564/89795/1378/5788/7538	15
BP		GO:0034135	regulation of toll-like receptor 2 signaling pathway	9/3375	12/18870	2.41816873259544e-05	0.0367689447889173	0.0350009391068565	1535/7128/730249/64127/9867/114609/9258/7096/10333	9
BP		GO:0034134	toll-like receptor 2 signaling pathway	11/3375	17/18870	2.51640097629239e-05	0.0367689447889173	0.0350009391068565	1535/79155/7128/3654/730249/64127/9867/114609/9258/7096/10333	11
BP		GO:0051090	regulation of DNA-binding transcription factor activity	105/3375	406/18870	3.28244915502979e-05	0.0367689447889173	0.0350009391068565	6872/22954/1540/55859/602/93649/8322/22976/3169/338699/4921/7804/8995/79092/55906/83737/140/7037/200186/5292/5621/84868/50943/7546/4838/64170/59307/60401/367/4793/7128/23028/3654/3586/6608/149951/79918/730249/3606/5314/100533105/23678/9641/5716/7158/3815/4615/10738/8807/6376/25950/58509/306/6280/150372/64127/959/5727/5610/4794/4914/190/8928/1649/8320/6911/117854/5987/7334/4825/8887/107/409/2033/84875/8945/56849/3397/114609/8553/55367/10892/7124/5925/8792/6737/7984/222487/285315/1440/4761/655/57121/6283/80279/5988/10333/26471/137735/9684/9314/3728/5336/9181/65992	105
BP		GO:0061008	hepaticobiliary system development	46/3375	145/18870	3.66578007890298e-05	0.0367689447889173	0.0350009391068565	10,269/5914/2487/5106/4851/55036/3482/5009/4838/2002/54658/54575/54577/54576/54600/7128/123872/3586/9821/10419/6648/23322/55959/2627/26257/5727/8928/6927/3720/51733/83606/7124/10370/9104/11231/34/7494/54903/80279/6928/2735/8140/3184/25836/5253/6926	46
BP		GO:0000725	recombinational repair	54/3375	179/18870	3.9450655639696e-05	0.0367689447889173	0.0350009391068565	8295/348654/196528/5892/2072/8438/7515/158880/3014/23028/2074/2187/55317/78995/56160/65123/79677/9985/7158/115004/10524/7156/27343/56893/29128/7454/23347/23126/63979/64859/79000/387893/4172/375757/7334/57697/2521/64110/22891/5889/7517/254394/79035/675/26122/6119/64710/55183/25939/254225/25836/6118/3364/79728	54
BP		GO:0000724	double-strand break repair via homologous recombination	53/3375	175/18870	4.11812181635874e-05	0.0367689447889173	0.0350009391068565	8295/348654/196528/5892/2072/8438/7515/158880/3014/23028/2074/2187/55317/56160/65123/79677/9985/7158/115004/10524/7156/27343/56893/29128/7454/23347/23126/63979/64859/79000/387893/4172/375757/7334/57697/2521/64110/22891/5889/7517/254394/79035/675/26122/6119/64710/55183/25939/254225/25836/6118/3364/79728	53
BP		GO:0007249	canonical NF-kappaB signal transduction	81/3375	300/18870	5.28138518853611e-05	0.0394248437513385	0.037529131264432	330/22954/6574/387/602/84336/356/554/6363/6366/57162/8904/79753/7037/5187/64170/57153/60401/4793/10758/79594/79155/7128/245937/55870/3654/389/338339/9641/4615/51523/541468/10738/6376/81858/58509/64127/54469/7098/841/93978/10783/55179/9020/10133/4055/684/84270/117854/7334/79671/6284/27032/56848/51311/409/28511/23636/8945/114609/5536/170506/55367/10892/7124/6737/8454/6093/6285/84292/51284/6283/5988/9516/6275/10333/284/4790/7105/5336/65992	81
BP		GO:0042113	B cell activation	76/3375	278/18870	5.67717750019274e-05	0.0394248437513385	0.037529131264432	975/5395/2213/602/22976/165918/1316/3446/6778/4773/57162/3467/7037/3449/8503/3447/3440/50943/2874/4323/3635/10758/79155/7128/1137/3516/3623/6777/3586/54440/4332/3443/3439/3445/7158/3815/3448/3451/23228/259307/10312/150372/64127/959/5074/6461/5585/841/4914/5580/343/684/100141515/2033/9984/114609/7163/51010/10892/1378/359948/64332/2355/11314/55183/3087/222487/5788/939/7494/27242/3567/112616/1141/1026/5336	76
CC		GO:0005925	focal adhesion	111/3520	421/19886	4.93469978870809e-06	0.00338833886578312	0.0031816066343954	6840/7529/975/387/7041/6385/4921/2195/10787/7430/811/3689/6202/5058/3655/5754/3693/3482/4323/5595/6207/3309/4162/998/71/54914/2321/867/126353/8394/4179/58497/120534/118/5094/64098/5880/2580/6278/6522/57514/10971/7052/6203/89/55827/374872/26986/7534/23164/7094/8462/11135/88/57477/6235/2317/6175/5329/3987/6237/10298/8573/11078/3603/5786/6130/9590/9564/2314/10094/6146/6136/57669/375/84951/3936/79038/6645/102/6181/6133/7791/50507/81/23114/3897/7145/83692/22919/5788/23122/567/2037/10544/23385/4008/6730/57175/6641/23545/79853/91179/9902/25996/3728/928/9181/9260/823/23768	111
CC		GO:0030055	cell-substrate junction	112/3520	431/19886	9.14531407768724e-06	0.00338833886578312	0.0031816066343954	6840/7529/975/387/7041/6385/4921/2195/10787/7430/811/3689/6202/5058/3655/5754/3693/3482/4323/5595/6207/3309/4162/998/71/54914/2321/867/126353/8394/4179/58497/120534/118/5094/64098/5880/2580/6278/6522/57514/10971/7052/6203/89/55827/374872/26986/7534/23164/7094/8462/11135/88/57477/6235/2317/6175/5329/3987/6237/10298/8573/11078/3603/5786/6130/9590/9564/2314/10094/6146/6136/57669/375/84951/3936/79038/6645/102/6181/6133/7791/50507/81/23114/3897/7145/83692/22919/5788/23122/567/2037/10544/23385/4008/6730/57175/6641/23545/23676/79853/91179/9902/25996/3728/928/9181/9260/823/23768	112
CC		GO:0000786	nucleosome	44/3520	134/19886	1.60871781166943e-05	0.00397353299482348	0.00373109639829295	8295/8369/8357/8360/8358/257218/8339/8345/8969/3006/8348/8362/8968/3010/8354/3014/7994/3008/55766/8351/7052/85236/10524/3662/8346/8294/54737/8340/8366/8364/8363/56848/440689/8342/26122/3015/64431/85235/23522/8355/7141/3012/3018/8970	44
CC		GO:0098687	chromosomal region	98/3520	399/19886	0.000307165683354358	0.0462225969107251	0.0434024242602305	1639/29117/545/54433/1786/8360/54108/9183/79980/196528/55920/5892/283489/81930/2072/1059/57459/3181/5515/5499/115426/92822/8362/7515/55355/1263/5424/3014/23028/5119/79902/56160/10734/25909/51490/79677/9985/5314/7158/10524/5048/5905/5518/8294/10111/23347/9735/11258/5001/8366/1063/8850/64859/25983/8364/79577/6602/81620/4172/54069/1060/10428/5976/8363/56897/991/4998/56155/7517/9984/79035/6847/675/1062/170506/23353/11004/11243/79682/55183/10664/7514/23122/3104/151246/57132/27085/57082/3012/9031/6118/5253/3018/6839/8970/5347/25936/84516	98
CC		GO:0036064	ciliary basal body	49/3520	172/19886	0.00031189336646913	0.0462225969107251	0.0434024242602305	1639/10013/1540/11092/7430/164395/5692/84080/6792/257177/643037/10807/85302/11190/8556/56912/5314/23322/2733/29070/353116/583/1063/27152/55717/55212/196383/9786/9867/1069/83547/27077/26005/283726/118491/79960/26146/84140/89891/22919/124590/158297/54903/729440/26259/95681/5578/755/25886	49
MF		GO:0140297	DNA-binding transcription factor binding	124/3443	477/18496	3.50747471375936e-05	0.0430367147378274	0.0427911915078642	6872/22954/10413/55859/6256/5396/602/93649/338699/7041/8328/9969/84660/811/8013/8345/8856/1609/8904/200186/2648/5187/50943/115426/10766/5595/2002/11176/8861/2623/7391/367/8638/7290/23028/64324/3516/7994/23019/6777/118/4209/6789/149951/23013/26043/79918/22984/7159/5705/51085/5716/7158/10524/51523/8022/468/7534/2627/4087/60491/10634/6879/583/4824/9611/55809/1387/5585/1063/190/10783/8850/3204/5978/8928/55212/79885/1649/8320/6911/2114/6880/117854/51339/4101/51564/2033/1869/84875/3301/1482/27063/1406/8553/10370/7508/5925/2353/171023/81/51053/1390/10865/11218/64710/10463/79447/3087/84792/59336/80139/26155/80279/9862/7110/1385/4780/9314/3728/8545/2932/6926/65992	124
**2,KEGG pathway enrichment analysis of the Conv1D**										
**category**	**subcategory**	**ID**	**Description**	**GeneRatio**	**BgRatio**	**pvalue**	**p.adjust**	**qvalue**	**geneID**	**Count**
Organismal Systems	Immune system	hsa04613	Neutrophil extracellular trap formation	62/1709	196/9521	1.98557527200052e-06	0.00069098019465618	0.000608213057002264	10,013/8369/1535/8357/23236/5332/8360/8358/2212/1184/8339/8345/3689/2214/8969/5894/110117499/8503/5595/8348/8362/8968/71/8354/3014/8331/5880/55766/8351/85236/8346/8294/4689/8340/8366/8364/79885/820/51564/6300/8363/83933/51311/2209/440689/8342/1378/79792/3015/85235/7419/8355/51284/3012/291/4790/5578/2215/3018/8970/7450/5336	62
Environmental Information Processing	Signal transduction	hsa04630	JAK–STAT signaling pathway	52/1709	168/9521	2.56063112410219e-05	0.00445549815593781	0.00392180872165125	598/3592/3446/149233/3595/133396/6778/282617/3467/5894/3449/5292/110117499/8503/3447/3440/55801/1271/3587/58985/3572/6777/4170/3586/29949/5156/4352/3443/3439/3445/9306/3448/1442/3451/1439/3953/1387/3455/5617/282616/10379/2033/5781/9180/3562/3590/1440/30837/3567/246778/50615/1026	52
Human Diseases	Infectious disease: bacterial	hsa05152	Tuberculosis	54/1709	182/9521	6.59030945166322e-05	0.00623072201831116	0.00548439233916681	387/2213/3592/5993/3446/2212/3689/2214/5894/3449/3929/3447/3440/5595/64170/1263/3587/3654/3920/3586/11151/1509/3606/3443/3439/3445/4615/3448/3451/51806/10312/64127/1387/841/8411/820/6300/56848/2209/2033/114609/10892/7124/1378/5994/23545/7096/10333/1385/9902/4790/2215/51606/7879	54
Environmental Information Processing	Signaling molecules and interaction	hsa04060	Cytokine-cytokine receptor interaction	80/1709	298/9521	7.16174944633467e-05	0.00623072201831116	0.00548439233916681	27,189/356/3592/3446/8995/149233/3595/133396/6363/6366/282617/3467/8784/3449/658/3447/3440/93/4838/55801/60401/1271/3587/58985/3623/3572/1233/3586/29949/269/353500/4352/3606/3443/3439/3445/3448/8807/1442/2920/6376/3451/1439/959/3953/3455/5617/282616/9210/2661/3603/4055/6370/151449/6372/10663/9180/7046/8771/285613/656/9966/944/7124/8792/4804/3562/3590/5197/6362/10563/1440/939/27242/655/3567/4982/4283/246778/50615	80
Human Diseases	Immune disease	hsa05322	Systemic lupus erythematosus	44/1709	144/9521	0.000145511255361465	0.010127583373158	0.00891447901267082	8369/8357/8360/8358/2212/8339/8345/941/2214/8969/8348/8362/8968/8354/3014/8331/3586/55766/8351/85236/8346/8294/959/8340/8366/8364/8363/2209/440689/729/8342/7124/712/6737/81/3015/85235/8355/3012/721/100293534/2215/3018/8970	44
Human Diseases	Cancer: overview	hsa05203	Viral carcinogenesis	57/1709	205/9521	0.000287999651333598	0.0167039797773487	0.0147031400943995	10,013/8369/7529/387/1019/5933/8360/8339/8345/2648/110117499/8503/5595/8348/8362/998/2966/730394/3516/3572/6777/1233/5423/10971/85236/8346/468/7534/8294/8340/5610/1387/841/8366/8850/8364/10379/79885/4055/51564/8363/83933/1027/991/440689/2033/8342/5925/81/728340/7419/1385/4790/1026/3018/8970/23352	57
Environmental Information Processing	Signal transduction	hsa04350	TGF-beta signaling pathway	34/1709	110/9521	0.000629924036831327	0.0313162235453288	0.0275650969500626	387/7018/5933/7036/7037/658/5515/93/4838/5595/79639/269/353500/23592/60436/5518/4087/9611/1387/6199/151449/2033/7046/3397/656/7124/9765/8454/6093/64750/4091/655/1875/5519	34
Environmental Information Processing	Signal transduction	hsa04151	PI3K-Akt signaling pathway	89/1709	362/9521	0.00076349884028945	0.0332121995525911	0.0292339687531882	9965/7529/1298/6256/2277/598/10110/1019/356/6194/7248/3446/5106/80310/3655/200186/5894/3326/3449/110117499/8503/3693/3447/3440/3911/5515/256076/5595/5528/2321/7422/1292/7143/4170/5156/2792/10971/3667/3443/3439/57818/100533105/23678/3445/3815/3448/253314/1442/3451/468/7534/5518/57521/4902/6199/5585/3455/5617/4914/55844/2997/4254/2251/23035/131873/2264/2253/1288/117145/1027/7424/2255/9180/3481/4846/4804/3562/51764/1440/26291/57121/1385/284/4790/1026/5578/5519/7450/2932	89
Organismal Systems	Immune system	hsa04620	Toll-like receptor signaling pathway	33/1709	109/9521	0.0011194660212048	0.0432860194865858	0.0381011242304793	3592/3446/941/3449/3929/110117499/8503/3447/3440/5595/3654/3443/3439/3445/9641/4615/3448/3451/7098/841/3455/10379/6300/51311/114609/7124/2353/5608/51284/7096/10333/4283/4790	33
Cellular Processes	Cell growth and death	hsa04110	Cell cycle	44/1709	158/9521	0.00130785915584101	0.0445136618096855	0.0391817168379265	545/7529/9587/1019/5933/29945/51433/5515/5528/8556/10971/994/902/494551/7534/5518/4087/51529/1387/5001/81620/4172/995/1027/991/2033/1869/4998/7029/51343/5925/8454/80174/23594/85417/27085/57082/1026/25836/1875/5519/168448/2932/5347	44
Human Diseases	Infectious disease: viral	hsa05161	Hepatitis B	45/1709	163/9521	0.0014070410342142	0.0445136618096855	0.0391817168379265	7529/356/4318/3446/6778/4773/5894/3449/110117499/8503/3447/3440/4214/5595/2002/3654/6777/10971/6554/3443/3439/3445/9641/4615/3448/3451/468/7534/7098/1387/841/6300/2033/1869/7046/114609/7124/5925/2353/7419/5608/1385/4790/1026/5578	45
Environmental Information Processing	Signal transduction	hsa04015	Rap1 signaling pathway	55/1709	212/9521	0.00214193606398242	0.0614880932369095	0.054122913284757	999/9965/387/2277/23236/5332/80310/3689/5894/110117499/8503/5595/998/7410/71/2321/84612/2902/7422/2775/5156/5880/3815/51806/7094/889/6237/4254/83593/2251/2264/50855/9855/2253/9564/6300/107/5028/7424/2255/6494/3397/5881/4804/3937/23094/64411/9002/5608/26291/11069/57121/27040/284/5578	55
Human Diseases	Endocrine and metabolic disease	hsa04931	Insulin resistance	32/1709	109/9521	0.00229696900022938	0.0614880932369095	0.054122913284757	11,000/5524/5106/10062/200186/6197/10724/110117499/8503/5499/5837/376497/5792/3667/10999/57818/51085/22877/6199/2997/5580/11001/9945/5565/5781/5507/7124/4846/5564/1385/4790/2932	32
Metabolism	Carbohydrate metabolism	hsa00040	Pentose and glucuronate interconversions	14/1709	36/9521	0.0024949595631183	0.0620175662832264	0.0545889648772501	54,658/54575/54659/54657/54579/54578/54577/54576/54600/6652/2990/7360/51181/55277	14
Human Diseases	Infectious disease: viral	hsa05171	Coronavirus disease - COVID-19	60/1709	238/9521	0.00284872327072552	0.066090379880832	0.0581739278442895	7311/6194/3592/3446/2212/6202/3449/110117499/8503/3447/3440/4142/5595/6230/6207/4793/4314/3654/3572/9045/3443/6192/3439/3445/9641/6203/51187/4615/115004/3448/3451/6235/7098/103/5610/6175/3455/10379/6130/10747/6146/6300/51311/6157/6136/729/6181/6133/7124/712/2353/6227/1440/51284/721/100293534/4790/5578/7450/5336	60
Human Diseases	Cardiovascular disease	hsa05417	Lipid and atherosclerosis	55/1709	216/9521	0.00331850961694038	0.0721775841684533	0.0635319933243191	6256/387/1535/598/356/3592/23236/5332/4318/3446/4773/3326/3449/3929/110117499/8503/3447/3440/6257/5595/1558/3309/998/7410/4314/3654/6648/3606/3443/3439/3445/9641/4615/3448/2920/3451/468/51806/4689/959/841/1649/6300/114609/7124/4846/2081/2353/7494/5608/10333/4780/4790/5578/2932	55
Organismal Systems	Immune system	hsa04650	Natural killer cell mediated cytotoxicity	37/1709	134/9521	0.00356406810144224	0.0729585705471706	0.064219431425368	356/3446/4773/3689/5058/2214/5894/3449/110117499/8503/3447/3440/5595/7410/51744/5880/3443/3439/3445/3448/3451/3455/6452/5781/7124/5881/3937/3802/3803/3804/100132285/3808/27040/5578/2215/3932/5336	37
Organismal Systems	Immune system	hsa04660	T cell receptor signaling pathway	34/1709	122/9521	0.00433103904613155	0.0837334215585434	0.0737036469253966	387/1019/5062/4773/5058/5894/110117499/8503/5515/5595/998/7410/4793/5528/3586/5518/959/4794/55844/10298/9020/6300/5781/10892/7124/2353/3937/5788/3567/27040/4790/3932/5519/2932	34
Human Diseases	Substance dependence	hsa05034	Alcoholism	49/1709	191/9521	0.00459607326522146	0.0841807103314246	0.074097358458695	10,013/8369/8357/8360/8358/8339/8345/8969/5894/5499/5595/8348/8362/8968/8354/3014/2902/2775/6531/8331/2792/55766/8351/85236/8346/468/51806/8294/8340/8366/8364/79885/1392/51564/8363/83933/2030/440689/8342/3015/2905/85235/8355/51764/84152/3012/1385/3018/8970	49
Metabolism	Carbohydrate metabolism	hsa00053	Ascorbate and aldarate metabolism	12/1709	31/9521	0.00521903888787691	0.0908112766490583	0.0799337008616938	54,658/54575/54659/54657/54579/54578/54577/54576/54600/219/2990/9104	12
Genetic Information Processing	Replication and repair	hsa03420	Nucleotide excision repair	20/1709	63/9521	0.00559159127480614	0.0926606554110733	0.0815615569407814	5426/2072/5981/2966/730394/5435/5424/2074/8450/57654/902/1069/7508/5427/3978/728340/6119/1161/4331/6118	20
**3,GO pathway enrichment analysis of the RFC**										
**ONTOLOGY**		**ID**	**Description**	**GeneRatio**	**BgRatio**	**pvalue**	**p.adjust**	**qvalue**	**geneID**	**Count**
BP		GO:0034329	cell junction assembly	81/1896	446/18870	9.37201588110588e-08	0.000545638764597984	0.000525523458933168	89,780/4208/222256/7057/389941/23229/56288/1005/28513/10391/3556/84612/1501/10395/1813/351/5728/5911/7481/57669/3273/10718/56940/10686/596/28316/1500/9693/94030/91862/387804/158038/575/8777/577/7010/10207/53942/2047/79983/84189/11141/5789/92737/60437/57502/324/2895/4131/1006/2824/23077/114798/395/26050/2889/79953/7368/22829/5818/55203/27253/1003/1812/257194/6616/3673/7058/149461/140689/287/9948/83700/5317/2596/100506658/84628/3915/2890/1002/2258	81
BP		GO:0055074	calcium ion homeostasis	61/1896	331/18870	2.15513395675874e-06	0.00627359494812468	0.00604231504613357	309/1258/196541/27069/8600/783/6352/729230/10523/2811/5023/489/3358/3356/2835/1813/351/1908/6262/9472/491/596/392862/8048/55177/1193/3062/6444/666/344905/817/2915/84239/65266/8614/25769/1756/664/55013/5144/5910/3357/1003/1812/23236/100507043/287/525/7224/1816/2149/662/2898/54499/845/5336/57620/2890/79572/6375/2280	61
BP		GO:0006874	intracellular calcium ion homeostasis	57/1896	308/18870	4.01740705652711e-06	0.00779644796103361	0.00750902715442804	309/1258/196541/27069/783/6352/729230/10523/2811/5023/489/3358/3356/2835/1813/351/1908/6262/9472/491/596/392862/8048/55177/1193/3062/666/344905/817/2915/84239/8614/25769/1756/664/55013/5144/5910/3357/1003/1812/23236/100507043/287/7224/1816/2149/662/2898/54499/845/5336/57620/2890/79572/6375/2280	57
BP		GO:0007416	synapse assembly	41/1896	200/18870	7.12167550269801e-06	0.010365598694177	0.00998346457970324	89,780/4208/389941/23229/3556/1813/351/5728/5911/10718/94030/387804/158038/575/577/53942/2047/84189/11141/5789/92737/57502/2895/4131/23077/114798/26050/79953/22829/5818/55203/27253/1812/257194/6616/7058/140689/2596/84628/2890/2258	41
BP		GO:0050808	synapse organization	78/1896	483/18870	1.64579812446193e-05	0.0191636733612348	0.018457192861071	1432/57689/89780/4208/389941/728/783/9499/23229/3556/1501/23181/440915/5621/1813/351/5728/5911/66000/10718/23657/94030/387804/8828/158038/575/23237/58504/5063/9758/577/53942/4009/91851/2047/84189/11141/5789/92737/158866/4774/57502/2895/4131/1488/11122/1287/2915/1006/55906/85461/23077/114798/26050/79953/22829/91752/5818/55203/27253/1812/257194/8898/6616/7058/27145/140689/2149/51104/3480/2596/3587/2064/153090/84628/57497/2890/2258	78
BP		GO:0099172	presynapse organization	16/1896	52/18870	3.11258468958062e-05	0.0281587840831912	0.0271206933430653	89,780/728/3556/351/5728/94030/53942/84189/11141/5789/57502/114798/26050/22829/27253/84628	16
BP		GO:0099054	presynapse assembly	15/1896	47/18870	3.38563188908174e-05	0.0281587840831912	0.0271206933430653	89,780/3556/351/5728/94030/53942/84189/11141/5789/57502/114798/26050/22829/27253/84628	15
BP		GO:0007409	axonogenesis	72/1896	448/18870	4.03115967291846e-05	0.0293367645196641	0.0282552468126798	80,031/57689/89780/2297/9499/56288/4692/4684/6696/84612/5362/6285/440915/1813/25942/351/5728/1908/284656/9706/7976/8301/596/8828/1785/575/3800/203228/5063/6586/3730/85440/53942/8239/4009/2047/1600/64283/84189/9355/57453/9353/4131/10494/23077/133418/114798/4781/26050/3670/4825/5818/655/4853/6616/26999/474/7224/673/3320/1947/3480/10716/2596/886/2064/84628/2737/9334/170302/1002/2258	72
BP		GO:0070588	calcium ion transmembrane transport	59/1896	352/18870	5.90456823508165e-05	0.0381959958496059	0.0367878771792748	7416/309/89780/196541/130507/27069/931/9254/783/10368/10523/2811/5023/489/5664/24145/3358/3356/116444/5621/1813/6262/9472/59284/491/117144/773/596/2906/5533/1193/140803/117532/4988/817/777/6332/25769/1756/5142/55013/80036/5144/3357/117531/6335/1812/23236/2332/287/7224/2149/662/54499/845/5336/57620/6375/2280	59
CC		GO:0097060	synaptic membrane	73/1977	393/19886	1.08110831986386e-07	7.23261465988922e-05	6.81667245893107e-05	57,689/51107/10368/1145/5023/3756/5664/23345/3356/22986/8572/116444/6511/1813/5911/59284/8301/491/94030/392862/8828/2906/1785/23237/57451/57706/577/22999/53942/57537/1144/1146/2892/401190/11141/22997/56479/5789/348980/57502/2742/2895/11122/2915/1006/55906/114798/1740/1756/79953/3760/1741/8573/22829/57692/5818/27253/1812/8898/2332/6616/287/115548/2149/51104/53826/10159/2898/2064/84628/57497/2890/9829	73
CC		GO:0014069	postsynaptic density	61/1977	315/19886	2.69337701618193e-07	9.00934611912856e-05	8.49122543522619e-05	57,689/23043/6711/10368/23229/3756/9922/2171/1501/22986/116444/54487/5621/5728/66000/59284/491/1500/94030/339451/392862/2906/6207/1785/575/23237/5063/8777/577/6790/9743/57537/2892/401190/22997/158866/57502/2895/4131/80315/11122/2915/85461/114798/1740/5142/664/79953/5071/1741/22829/120/8898/2332/23705/51104/2596/2898/100131897/2890/9829	61
CC		GO:0045211	postsynaptic membrane	54/1977	275/19886	8.16387333388248e-07	0.000144892020200749	0.000136559389662889	57,689/10368/1145/5023/3756/23345/3356/22986/8572/116444/1813/59284/8301/491/94030/392862/8828/2906/1785/23237/57451/577/57537/1144/1146/2892/401190/11141/22997/348980/57502/2742/2895/11122/2915/55906/114798/1740/1756/79953/1741/22829/57692/27253/1812/2332/287/2149/51104/53826/10159/2898/57497/2890	54
CC		GO:0098984	neuron to neuron synapse	66/1977	362/19886	8.66320001200295e-07	0.000144892020200749	0.000136559389662889	57,689/23043/389941/6711/10368/23229/3756/9922/143425/2171/1501/22986/116444/54487/5621/5728/66000/59284/491/1500/94030/339451/392862/2906/6207/1785/575/23237/5063/8777/577/6790/9743/57537/2892/401190/22997/5789/158866/57502/2895/4131/80315/11122/2915/85461/114798/1740/5142/664/79953/5071/1741/22829/120/5818/8898/2332/23705/51104/2596/2898/100131897/9465/2890/9829	66
CC		GO:0032279	asymmetric synapse	61/1977	331/19886	1.54169182033803e-06	0.00017795959043905	0.000167725268937127	57,689/23043/6711/10368/23229/3756/9922/2171/1501/22986/116444/54487/5621/5728/66000/59284/491/1500/94030/339451/392862/2906/6207/1785/575/23237/5063/8777/577/6790/9743/57537/2892/401190/22997/158866/57502/2895/4131/80315/11122/2915/85461/114798/1740/5142/664/79953/5071/1741/22829/120/8898/2332/23705/51104/2596/2898/100131897/2890/9829	61
CC		GO:0099572	postsynaptic specialization	63/1977	346/19886	1.59605013846681e-06	0.00017795959043905	0.000167725268937127	57,689/23043/6711/10368/23229/3756/9922/2171/1501/22986/116444/54487/5621/5728/66000/59284/491/1500/94030/339451/392862/2906/6207/1785/575/23237/5063/8777/577/6790/9743/57537/1144/2892/401190/22997/158866/57502/2895/4131/80315/11122/2915/85461/114798/1740/5142/664/79953/5071/55914/1741/22829/120/8898/2332/23705/51104/2596/2898/100131897/2890/9829	63
CC		GO:0098839	postsynaptic density membrane	26/1977	99/19886	2.8029848067299e-06	0.000267885262243186	0.000252479383343039	57,689/10368/3756/22986/116444/59284/491/94030/2906/23237/577/57537/2892/401190/57502/2895/11122/2915/114798/1740/79953/1741/22829/51104/2898/2890	26
CC		GO:0005912	adherens junction	38/1977	188/19886	1.67951911794973e-05	0.00140449786238546	0.00132372625217354	27,295/222256/8613/56288/83692/83461/1005/28513/54800/1501/8572/29964/57669/55966/247/28316/1500/117583/1525/9414/357/79983/60437/83478/324/1006/23650/1740/182/1741/5818/1003/5317/301/5239/23336/55691/1002	38
CC		GO:0099634	postsynaptic specialization membrane	27/1977	122/19886	5.31770329208233e-05	0.00395282611378119	0.00372550207246469	57,689/10368/3756/22986/116444/59284/491/94030/2906/23237/577/57537/1144/2892/401190/57502/2895/11122/2915/114798/1740/79953/1741/22829/51104/2898/2890	27
CC		GO:0034703	cation channel complex	36/1977	193/19886	0.000156684664984685	0.00945146280682661	0.00890791632647178	126,755/1258/9254/783/10368/7881/3754/3756/57657/6262/9472/59284/117144/93107/10060/773/3763/257062/80333/3762/26251/56479/348980/777/6332/5142/55013/5144/3760/6335/1260/7224/2898/845/6833/2280	36
CC		GO:0043025	neuronal cell body	74/1977	489/19886	0.000163316873265462	0.00945146280682661	0.00890791632647178	9499/4692/729230/58157/54800/1728/3756/5664/11075/3356/57657/1501/2695/116444/6581/6285/1813/80975/351/59284/23245/6515/7976/8301/491/445/1536/773/6860/9693/339451/23237/3800/203228/6790/5126/80333/57537/401190/1393/92737/4988/3672/1620/4131/777/57282/345193/7150/55906/85461/1740/1996/23001/6653/91752/339479/2690/257194/2332/6616/57626/51218/5562/7224/8507/3320/3480/2596/2898/65109/153090/60481/2890	74
CC		GO:0042734	presynaptic membrane	32/1977	165/19886	0.000169532965144872	0.00945146280682661	0.00890791632647178	51,107/5023/3756/5664/3356/6511/1813/8301/491/94030/2906/57706/22999/53942/401190/56479/5789/348980/3760/8573/5818/27253/1812/2332/6616/115548/53826/2898/2064/84628/2890/9829	32
CC		GO:0005769	early endosome	65/1977	427/19886	0.000336487276560193	0.0173161529245207	0.0163203140615025	1238/9100/23041/10239/51107/55198/89780/125058/4430/913/753/3756/5664/3105/81609/3135/8572/351/11156/66000/59284/23245/8301/3833/11031/23516/10938/7251/23237/57705/57537/2047/1183/666/92737/1730/84239/9910/121260/8905/10228/399979/2889/79953/51617/3140/6653/23317/55614/8898/6456/287/84868/10269/2149/55435/301/286410/2150/2064/51554/2890/112936/79572/9711	65
CC		GO:0098978	glutamatergic synapse	62/1977	407/19886	0.000447031457929247	0.0213617175253333	0.0201332213007233	1432/57689/89780/23043/389941/6711/10368/5023/3556/3356/81609/22986/6511/54487/1813/59284/10718/491/1500/6860/93664/94030/339451/392862/8828/2906/5573/5533/23237/58504/5063/577/6790/2047/401190/11141/5789/348980/57502/2895/1488/11122/2915/1006/85461/114798/1996/55914/1741/22829/27253/1812/23236/6616/6456/1977/10082/27145/53826/2898/84628/2890	62
CC		GO:0016342	catenin complex	10/1977	31/19886	0.000576102851011488	0.0256941871551124	0.0242165338776057	222,256/1005/28513/28316/1500/60437/324/1006/1003/1002	10
CC		GO:0034704	calcium channel complex	18/1977	79/19886	0.000617379768891008	0.0258141915867553	0.0243296369451127	9254/783/10368/6262/9472/59284/117144/773/257062/777/6332/5142/55013/5144/6335/7224/845/2280	18
CC		GO:0043204	perikaryon	28/1977	149/19886	0.000708910846665239	0.0278977268481791	0.0262933496688841	4692/729230/58157/54800/3756/1501/1813/351/23245/6515/445/203228/5126/57537/401190/1393/4988/3672/4131/57282/345193/7150/1740/1996/23001/2332/2596/2898	28
CC		GO:0045177	apical part of cell	68/1977	469/19886	0.000967207586965833	0.0359478819822301	0.0338805464673997	947/479/115584/23043/728/134288/4430/56288/960/5664/60676/3570/84612/6583/9497/10456/6581/6579/28232/55630/5420/351/5728/349633/10913/2195/90/7976/528/55966/23657/491/9693/117583/23516/8842/8777/7010/140803/84552/4646/10207/357/57710/2028/56606/57282/286204/4935/182/5144/1896/92521/9962/2191/287/525/5562/5593/55/131578/3320/301/100506658/3587/2064/64116/10083	68
CC		GO:0034702	monoatomic ion channel complex	47/1977	300/19886	0.00114576772357454	0.0403430845827035	0.0380229842892603	126,755/1258/9254/783/10368/1145/7881/3754/3756/57657/116444/6262/9472/59284/1187/117144/93107/10060/773/3763/257062/2906/1193/80333/1144/2892/3762/26251/56479/348980/2742/2895/777/6332/5142/55013/5144/3760/1741/6335/1260/7224/2898/845/2890/6833/2280	47
CC		GO:0005923	bicellular tight junction	24/1977	126/19886	0.0013562327058533	0.0453659840107928	0.0427570205687435	26/56288/84612/10686/9693/91862/117583/1525/9414/8777/84552/10207/357/79983/324/65266/54587/1741/1003/149461/387597/83700/100506658/55691	24
MF		GO:0030695	GTPase regulator activity	83/1891	486/18496	1.88405411196192e-06	0.00105318624858671	0.00100251510904921	9411/57514/125058/23229/54974/57533/9922/3257/89781/10395/23550/10045/255426/3843/51195/10144/6002/5996/6000/9610/7481/58480/100271927/126432/10928/9462/55616/50619/7409/9693/9815/11033/729540/3156/8729/201627/58504/203228/57706/577/85440/1950/22999/9743/10788/1121/23179/64283/116984/139818/26575/23216/9353/57568/83478/9882/23637/9515/1794/55103/9910/23077/55296/395/5922/140885/26130/2889/57186/84364/5910/3357/9754/23236/60682/116987/54453/23677/93627/23365/55667/153090/9855	83
MF		GO:0060589	nucleoside-triphosphatase regulator activity	83/1891	486/18496	1.88405411196192e-06	0.00105318624858671	0.00100251510904921	9411/57514/125058/23229/54974/57533/9922/3257/89781/10395/23550/10045/255426/3843/51195/10144/6002/5996/6000/9610/7481/58480/100271927/126432/10928/9462/55616/50619/7409/9693/9815/11033/729540/3156/8729/201627/58504/203228/57706/577/85440/1950/22999/9743/10788/1121/23179/64283/116984/139818/26575/23216/9353/57568/83478/9882/23637/9515/1794/55103/9910/23077/55296/395/5922/140885/26130/2889/57186/84364/5910/3357/9754/23236/60682/116987/54453/23677/93627/23365/55667/153090/9855	83
MF		GO:0005096	GTPase activator activity	50/1891	272/18496	2.92132277949876e-05	0.010886796224932	0.0103630081756956	9411/57514/125058/57533/10395/10144/6002/5996/6000/9610/7481/100271927/126432/10928/9462/55616/9693/9815/11033/729540/58504/577/9743/10788/1121/116984/26575/23216/57568/83478/9882/23637/9515/1794/9910/55296/395/5922/26130/57186/84364/3357/9754/23236/60682/116987/54453/93627/23365/153090	50
MF		GO:0044325	transmembrane transporter binding	29/1891	133/18496	6.33722546498059e-05	0.0167035341727308	0.0158998898866687	126,755/7416/9100/10523/489/7881/51185/24145/5716/5621/285966/9472/23327/93107/10060/9414/22999/2257/817/5142/5144/1003/2332/6616/287/3320/6833/2280/2258	29
MF		GO:0001965	G-protein alpha-subunit binding	11/1891	29/18496	7.47027467474543e-05	0.0167035341727308	0.0158998898866687	1813/23328/5996/6000/4988/3357/1812/2149/3480/2150/2890	11
MF		GO:0008143	poly(A) binding	10/1891	27/18496	0.000200247709220392	0.0317808706890227	0.0302518221133622	9987/390748/79882/202559/1654/645974/340529/1996/5937/140886	10
MF		GO:0030552	cAMP binding	9/1891	23/18496	0.000257792846957203	0.0317808706890227	0.0302518221133622	1258/57657/9693/5573/10846/348980/5142/5144/1260	9
MF		GO:0005244	voltage-gated monoatomic ion channel activity	37/1891	200/18496	0.000264326071573915	0.0317808706890227	0.0302518221133622	126,755/7416/9254/783/10368/7881/3754/3756/57657/116444/59284/1187/117144/93107/1536/10060/773/3763/2906/1193/3758/80333/1183/3762/90134/26251/56479/117532/4988/348980/777/6332/3760/117531/6335/6616/6833	37
MF		GO:0022832	voltage-gated channel activity	37/1891	200/18496	0.000264326071573915	0.0317808706890227	0.0302518221133622	126,755/7416/9254/783/10368/7881/3754/3756/57657/116444/59284/1187/117144/93107/1536/10060/773/3763/2906/1193/3758/80333/1183/3762/90134/26251/56479/117532/4988/348980/777/6332/3760/117531/6335/6616/6833	37
MF		GO:0070717	poly-purine tract binding	11/1891	33/18496	0.000284265390778379	0.0317808706890227	0.0302518221133622	9987/390748/79882/202559/1654/645974/340529/1996/2332/5937/140886	11
MF		GO:0022843	voltage-gated monoatomic cation channel activity	30/1891	153/18496	0.000351884393572152	0.0357642501830606	0.0340435523350666	126,755/9254/783/10368/7881/3754/3756/57657/59284/117144/93107/10060/773/3763/2906/3758/3762/90134/26251/56479/117532/4988/348980/777/6332/3760/117531/6335/6616/6833	30
MF		GO:0022839	monoatomic ion gated channel activity	53/1891	325/18496	0.000419572450857082	0.038901201474594	0.0370295779030359	126,755/7416/309/1258/9254/783/10368/1145/5023/7881/3754/3756/57657/116444/6262/59284/1187/117144/93107/1536/10060/773/3763/441027/2906/1193/3758/80333/357/1183/1144/1146/2892/3762/90134/26251/56479/117532/4988/348980/2742/2895/777/6332/3760/117531/6335/6616/1260/662/2898/2890/6833	53
MF		GO:0022836	gated channel activity	53/1891	326/18496	0.000452339552030162	0.038901201474594	0.0370295779030359	126,755/7416/309/1258/9254/783/10368/1145/5023/7881/3754/3756/57657/116444/6262/59284/1187/117144/93107/1536/10060/773/3763/441027/2906/1193/3758/80333/357/1183/1144/1146/2892/3762/90134/26251/56479/117532/4988/348980/2742/2895/777/6332/3760/117531/6335/6616/1260/662/2898/2890/6833	53
MF		GO:1901981	phosphatidylinositol phosphate binding	33/1891	179/18496	0.000588359625690103	0.0469847186801096	0.0447241790656161	4430/56288/3756/143425/81609/56270/4689/8301/11033/8729/29916/55062/9758/57706/79109/3758/9743/10788/116984/695/139285/5321/55614/23236/55102/54843/115548/2596/115004/153090/79685/285590/9711	33
Human Diseases	Neurodegenerative disease	hsa05017	Spinocerebellar ataxia	30/921	144/9521	3.88238757252057e-05	0.0133554132494708	0.0115654282423507	7416/387332/489/116444/6511/5601/9706/10000/7494/773/2906/55062/6315/2892/1600/5704/5602/5295/115209/4976/6908/5707/5290/5713/10213/5695/23236/55102/5689/2890	30
Human Diseases	Infectious disease: viral	hsa05165	Human papillomavirus infection	52/921	333/9521	0.000324519391182036	0.0392772182527754	0.0340130133584316	6932/1978/89780/8313/7157/7057/56288/387332/284217/1285/256076/131873/6696/84612/3105/23493/3135/57801/5315/5728/1869/10000/7481/7976/528/1027/55534/1950/84552/10207/5523/3672/324/5295/1287/6908/1740/3918/182/1297/5290/1741/4853/3673/7058/525/8992/3845/5522/5734/3915/5527	52
Human Diseases	Cardiovascular disease	hsa05418	Fluid shear stress and atherosclerosis	27/921	142/9521	0.000449565157813773	0.0392772182527754	0.0340130133584316	1432/5603/4205/4208/7157/652/1728/221357/2940/6383/5601/90/10000/3554/445/596/4259/5602/5295/4688/1843/5290/1003/5562/3320/6401/92	27
Environmental Information Processing	Signal transduction	hsa04012	ErbB signaling pathway	19/921	86/9521	0.000456711840148551	0.0392772182527754	0.0340130133584316	1978/9542/5601/10000/10718/1027/5063/1950/57144/27/5602/817/5295/5290/3845/3084/673/5336/2064	19
Environmental Information Processing	Signaling molecules and interaction	hsa04512	ECM-receptor interaction	19/921	89/9521	0.000717172033937992	0.0431903268662005	0.037401660046312	7057/960/284217/2811/1285/256076/131873/6696/341640/22987/3672/1287/158326/3918/80144/1297/3673/7058/3915	19
Metabolism	Amino acid metabolism	hsa00270	Cysteine and methionine metabolism	13/921	52/9521	0.00105743651234008	0.0431903268662005	0.037401660046312	4548/3939/3948/1788/58478/29968/23743/160287/2729/4507/137362/2806/6611	13
Human Diseases	Drug resistance: antineoplastic	hsa01522	Endocrine resistance	20/921	99/9521	0.00109578373456348	0.0431903268662005	0.037401660046312	1432/5603/7157/1869/5601/10000/596/1870/1027/5602/5295/182/5290/4853/3845/673/3480/2064/1031/1029	20
Human Diseases	Cancer: specific types	hsa05213	Endometrial cancer	14/921	59/9521	0.00119657678579931	0.0431903268662005	0.037401660046312	6932/8313/7157/4292/5728/10000/1950/324/5295/51426/5290/3845/673/2064	14
Human Diseases	Cancer: specific types	hsa05222	Small cell lung cancer	19/921	93/9521	0.00125601492869514	0.0431903268662005	0.037401660046312	7157/284217/1285/5728/1869/10000/596/1870/1027/6258/5295/1287/51426/331/3918/5290/3673/7185/3915	19
Cellular Processes	Cellular community - eukaryotes	hsa04550	Signaling pathways regulating pluripotency of stem cells	26/921	144/9521	0.00126735916585317	0.0431903268662005	0.037401660046312	1432/5603/6932/89780/8313/652/3720/5013/80712/2103/90/10000/7481/7976/6929/324/5295/3670/5290/1852/3845/463/3480/9421/92/7547	26
Environmental Information Processing	Signaling molecules and interaction	hsa04514	Cell adhesion molecule (CAM) interaction	28/921	160/9521	0.00138108603351223	0.0431903268662005	0.037401660046312	57,689/947/4684/83692/2734/3105/3135/6383/10686/94030/1525/84189/5789/57502/6403/114798/26050/22829/5818/1003/257194/149461/23705/83700/6401/100506658/84628/1002	28
Cellular Processes	Cell growth and death	hsa04216	Ferroptosis	11/921	42/9521	0.00168411382141365	0.0467383488281779	0.0404741515250133	23,305/643246/7157/2180/5621/23657/1536/23516/2729/30061/64116	11
Environmental Information Processing	Signal transduction	hsa04066	HIF-1 signaling pathway	21/921	110/9521	0.00176627481036719	0.0467383488281779	0.0404741515250133	1978/3939/3948/3570/3098/2034/10000/2027/1536/596/1027/160287/1950/7010/817/5295/5290/1977/3480/5336/2064	21
NA	NA	hsa04517	IgSF CAM signaling	45/921	302/9521	0.00212154367625393	0.0521293589022395	0.0451426210811927	1432/5603/57689/7280/6711/56288/4684/3556/84612/5362/351/5601/10000/653857/7409/9693/94030/8828/1525/9414/5063/6586/8777/22999/84552/84189/11141/57144/5789/4068/57502/9353/10092/5602/5295/114798/26050/5290/8573/22829/5818/23705/287/83700/5336	45
Organismal Systems	Sensory system	hsa04750	Inflammatory mediator regulation of TRP channels	19/921	99/9521	0.00268875145668354	0.0616620334066092	0.0533976605081714	1432/5603/3556/3358/3356/5601/3827/3554/5602/817/5295/5290/3357/5321/23236/1573/5734/5336/2150	19
Human Diseases	Drug resistance: antineoplastic	hsa01521	EGFR tyrosine kinase inhibitor resistance	16/921	80/9521	0.00369477619676708	0.0750213147549594	0.0649664384199924	1978/9542/3570/5728/10000/596/1950/5295/5290/1977/3845/3084/673/3480/5336/2064	16
Human Diseases	Cancer: specific types	hsa05223	Non-small cell lung cancer	15/921	73/9521	0.00371863590170918	0.0750213147549594	0.0649664384199924	7157/1869/10000/1870/3800/1950/6258/5295/51426/5290/3845/673/5336/2064/1029	15
Human Diseases	Cancer: specific types	hsa05224	Breast cancer	25/921	148/9521	0.00395796345322492	0.0750213147549594	0.0649664384199924	6932/89780/8313/7157/8600/23493/5728/1869/10000/7481/7976/1870/4791/1950/324/5295/51426/182/5290/4853/3845/673/5241/3480/2064	25
Organismal Systems	Nervous system	hsa04728	Dopaminergic synapse	23/921	133/9521	0.00414361912890764	0.0750213147549594	0.0649664384199924	1432/5603/2784/1813/5601/10000/773/3763/5533/3800/5523/2892/3762/5602/817/3760/1812/23236/1816/5522/5527/2890/9575	23
Human Diseases	Cancer: specific types	hsa05214	Glioma	15/921	76/9521	0.00552771842315465	0.0937549787085195	0.0811892869477081	7157/5728/1869/10000/1870/1950/817/5295/51426/5290/3845/673/3480/5336/1029	15
Human Diseases	Cancer: specific types	hsa05215	Prostate cancer	19/921	106/9521	0.00588064945660464	0.0937549787085195	0.0811892869477081	6932/7157/5728/1869/10000/596/1870/1027/2119/2078/1950/5295/2915/5290/3845/673/3320/3480/2064	19
Environmental Information Processing	Signal transduction	hsa04024	cAMP signaling pathway	34/921	226/9521	0.00605191702163458	0.0937549787085195	0.0811892869477081	1258/5733/489/4852/2695/116444/1813/1908/5601/6262/10000/491/7409/2906/4772/10846/2892/2735/5602/817/5295/7434/5142/5144/486/5290/1812/1260/1816/2149/673/3350/2890/2737	34
Human Diseases	Cancer: specific types	hsa05212	Pancreatic cancer	15/921	77/9521	0.00626850148341845	0.0937549787085195	0.0811892869477081	7157/1869/5601/10000/10928/1870/1950/5602/5295/51426/5290/3845/673/2064/1029	15
Cellular Processes	Cellular community - eukaryotes	hsa04530	Tight junction	27/921	170/9521	0.00666313340277623	0.0944774658252172	0.0818149413357909	56,288/910/913/84612/51762/5601/653857/23327/10686/9693/91862/9414/2626/53632/8777/84552/10207/10092/5602/1740/1741/149461/5562/83700/5522/100506658/2064	27
Cellular Processes	Cellular community - eukaryotes	hsa04510	Focal adhesion	31/921	203/9521	0.00686609490008846	0.0944774658252172	0.0818149413357909	7057/284217/1285/256076/131873/6696/64098/5728/5601/10000/596/7409/5063/1950/57144/3672/5602/5295/1287/331/3918/2889/8395/1297/5290/3673/7058/673/3480/2064/3915	31

Cross-validation with the IntOGen Cancer Drivers database (v2024.09.20) [36] indicated that Conv1D-selected genes maintained slightly higher biological relevance compared to RFC. Among all selected genes (selection pval ≤0.05), 155/4329 (3.6%) of Conv1D genes overlapped with known cancer drivers, versus 90/2666 (3.4%) for RFC (Fisher's exact test, Conv1D versus RFC: *P* = .35), corresponding to a 6.1% relative enrichment gain over RFC. Although the difference was not statistically significant, these results suggest that Conv1D tends to select a broader and more biologically coherent set of genes.

These findings demonstrate that the Conv1D model's self-attention mechanism not only achieves higher classification accuracy but also prioritizes biologically meaningful, cancer-type-specific features over dataset-specific noise, outperforming traditional machine learning models in both predictive and functional relevance.

## Discussion

The integration of advanced machine learning techniques into cancer classification represents a significant advancement in precision oncology. Our self-attention based Conv1D network achieved an overall classification accuracy of 90.20%, with the highest accuracy observed in STAD (95.79%) and the lowest in CESC (77.46%). The high precision and recall rates underscore the model's ability to accurately distinguish between cancer types despite their genetic complexity. In a clinical setting, precise cancer classification is crucial for guiding treatment strategies. The inclusion of local data, such as BGI's 688 Panel sequencing data with clinical applicability, enhancing the model's practicality and reliability in clinical environments.

The hierarchical clustering analysis using the top 1000 genes identified by our model revealed genetic relationships between cancer types that are not apparent through traditional classifications. In the dendrogram resulting from cluster analysis, CESC and CHOL exhibit relative proximity in spatial distance, which corresponds to the phenomenon observed in the confusion matrix where 16.90% of CESC samples are misclassified as CHOL. Similarly, LIHC and CHOL demonstrate mutual misclassification, which aligns with their close positioning in the dendrogram. These observations suggest that cancer types with closer spatial distances have a higher probability of being mutually misclassified during the classification process. This conclusion becomes more evident in the hierarchical clustering analysis conducted using the top 1000 genes. This suggests that focusing on the most influential genes can provide a more precise understanding of cancer's molecular characteristics, potentially guiding more effective treatment strategies.

Comparing dendrograms reveals the impact of focusing on influential genes. The all-genes dendrogram captures wider genetic variation with larger distances and dispersed clustering [37]. In contrast, the top 1000 genes dendrogram shows tighter clustering with smaller distances, suggesting these genes better indicate genetic relationships between cancer types. The CESC-CHOL cluster in the top 1000 genes dendrogram reveals shared genetic features not apparent when considering all genes, implying these genes more significantly define these cancers' molecular characteristics. The consistent COAD-LIHC clustering supports their genetic similarity, while LUSC and OV clustering differences highlight distinct aspects of their genetic profiles revealed by the top genes.

The identification of key genes such as C3orf36, JHY, and TASP1, and their significant mutation patterns across different cancers, provides valuable insights into the genetic landscape of these diseases [38]. Comprehensive pathway analysis provided molecular insights into different biological processes that define cancer type identities. The enrichment analysis further elucidated the biological pathways associated with the high-weight genes in the classification model, including Acute myeloid leukemia and TNF signaling, which are known to play crucial roles in cancer development and progression [39, 40]. The high statistical significance of these findings supports their relevance in understanding cancer biology.

A critical distinction between the Conv1D model and traditional machine learning approaches lies in biological interpretability rather than marginal performance gains. While Random Forest achieved comparable discriminatory power (mean AUC: 0.9901 versus Conv1D's 0.9916), functional enrichment analysis revealed fundamental differences: Conv1D identified 60% more significantly stable genes (3776 versus 2353) enriched for cancer-type-specific pathways—"Hepatitis B″ (q = 0.064) capturing viral etiology in hepatocellular carcinoma, ‘Neutrophil extracellular trap formation’ (q = 0.0006) and "JAK–STAT signaling" (q = 0.004) representing pan-cancer oncogenic drivers. In stark contrast, Random Forest's top pathways comprised neuronal processes mechanistically irrelevant to epithelial tumors: "Spinocerebellar ataxia" (q = 0.012) and "Dopaminergic synapse" (q = 0.065), likely reflecting batch artifacts rather than causal biology. This divergence stems from algorithmic design: Random Forest's greedy node-splitting prioritizes statistical variance maximization regardless of biological plausibility, whereas Conv1D's self-attention mechanism—optimized end-to-end via gradient descent—forces the model to learn multi-gene patterns with genuine cross-validation discriminatory power. Cross-validation with IntOGen Cancer Drivers confirmed Conv1D's superior biological coherence (3.6% driver gene overlap versus Random Forest's 3.4%). For clinical deployment, this interpretability advantage is essential. A model attributing predictions to tissue-specific oncogenic pathways (e.g. viral integration in LIHC, inflammation in STAD) provides actionable therapeutic hypotheses.

While our model showed remarkable performance, certain limitations must be acknowledged. Cholangiocarcinoma and other cancers exhibit a slight overlap in classification. These misclassifications indicate that the model can be further improved to enhance differentiation between closely related cancer types.

Regarding gene importance in our classification model, we emphasize the distinction between computational feature attribution and biological causality. When certain genes are reported to "improve" classification, this refers to their discriminative utility in the learned feature space rather than implying causal driver roles. The self-attention mechanism provides model interpretability by computing sample-specific weights that quantify each gene's contribution to predictions. For example, TP53 receives high attention weights because its mutation frequency varies systematically across cancer types—frequently mutated in ovarian and colorectal cancers but rarely in thyroid cancers—providing discriminative signals the model exploits. Statistical validation confirms that high-weight genes exhibit significant inter-group mutation burden differences (Kruskal-Wallis, *P* < .001) and enrich for cancer-relevant pathways. However, high attention weight indicates predictive utility but not necessarily driver status, as genes may be important due to co-mutation patterns or as proxies for broader mutational processes. This distinction between predictive utility and biological mechanism is fundamental to proper interpretation, positioning feature importance as hypothesis-generating rather than conclusive evidence of gene function.

A key consideration in interpreting our results is the distinction between mutation-based and expression-based approaches to cancer classification. While both modalities have demonstrated utility, they address different clinical scenarios and capture complementary biological information. Expression-based models excel when high-quality RNA-seq data are available and can leverage the rich information content of transcriptional profiles. However, clinical reality often presents constraints that favor mutation-based approaches. Panel capture sequencing is more widely implemented in routine clinical practice than RNA-seq, particularly for FFPE archival tissues where RNA degradation limits expression profiling. Moreover, mutation profiles represent stable genomic alterations that persist across disease progression and are less susceptible to technical variability from sample handling or batch effects. For cancer type classification—where the goal is to identify stable molecular identity rather than transient cellular states—mutation patterns may be particularly informative, as cancer type-specific driver mutations are diagnostically specific at the DNA level. Our study demonstrates that mutation count features, despite lower information density than expression data, can achieve robust classification performance (accuracy: 0.902, AUC: 0.990) using data routinely generated in clinical settings. This positions mutation-based classification as a valuable complementary approach that extends molecular diagnostics to scenarios where expression data are unavailable or impractical to obtain.

Additionally, the reliance on panel capture sequencing data, although more clinically applicable, may miss some nuances captured by whole-exome sequencing. Future studies should explore the integration of both sequencing methods to enhance the model's robustness.

## Conclusion

In conclusion, our self-attention based Conv1D network offers a powerful tool for cancer classification, leveraging genomic data to improve diagnostic accuracy and treatment planning. The identification of key genes and significant mutations, coupled with pathway enrichment, provides a comprehensive framework for understanding cancer biology and advancing precision oncology. Further validation and refinement of this approach could significantly impact clinical practice, enhancing patient care and outcomes.

**Key Points**

- Developed a self-attention based Conv1D network for cancer classification using panel capture sequencing data with >90% overall accuracy
- Identified key discriminative genes including C3orf36, JHY, and TASP1 that show significant mutation pattern differences between cancer types
- Demonstrated robust performance across diverse cancer types, with high recall rates despite the molecular heterogeneity challenges of tumors

## Supplementary Material

Supplementary_Table_bbag120

## Data Availability

The data used in this study were compiled from multiple sources, including previously published datasets and additional datasets that are part of ongoing or unpublished studies. Publicly available data can be accessed from the original publications as cited in this manuscript [41–44]. Due to ethical and data-sharing restrictions associated with the unpublished datasets, these data are not publicly available but may be obtained from the corresponding author upon reasonable request and subject to relevant approvals.

## References

[1] Tsimberidou AM . Targeted therapy in cancer. *Cancer Chemother Pharmacol* 2015;76:1113–32. 10.1007/s00280-015-2861-1 26391154 PMC4998041

[2] Gubin MM, Zhang X, Schuster H et al. Checkpoint blockade cancer immunotherapy targets tumour-specific mutant antigens. *Nature* 2014;515:577–81. 10.1038/nature13988 25428507 PMC4279952

[3] Albert FW, Kruglyak L. The role of regulatory variation in complex traits and disease. *Nat Rev Genet* 2015;16:197–212. 10.1038/nrg3891 25707927

[4] Schaub MA, Boyle AP, Kundaje A et al. Linking disease associations with regulatory information in the human genome. *Genome Res* 2012;22:1748–59. 10.1101/gr.136127.111 22955986 PMC3431491

[5] Vogelstein B, Kinzler KW. Cancer genes and the pathways they control. *Nat Med* 2004;10:789–99. 10.1038/nm1087 15286780

[6] Lawrence MS, Stojanov P, Polak P et al. Mutational heterogeneity in cancer and the search for new cancer-associated genes. *Nature* 2013;499:214–8. 10.1038/nature12213 23770567 PMC3919509

[7] Hanahan D, Weinberg RA. Hallmarks of cancer: the next generation. *Cell* 2011;144:646–74. 10.1016/j.cell.2011.02.013 21376230

[8] Chin L, Gray JW. Translating insights from the cancer genome into clinical practice. *Nature* 2008;452:553–63. 10.1038/nature06914 18385729 PMC2730524

[9] Chin L, Andersen JN, Futreal PA. Cancer genomics: from discovery science to personalized medicine. *Nat Med* 2011;17:297–303. 10.1038/nm.2323 21383744

[10] Mardis ER . The impact of next-generation sequencing on cancer genomics: from discovery to clinic. *Cold Spring Harb Perspect Med* 2019;9:a036269. 10.1101/cshperspect.a036269 PMC6719592 30397020

[11] Gibbs SN, Peneva D, Cuyun Carter G et al. Comprehensive review on the clinical impact of next-generation sequencing tests for the Management of Advanced Cancer. *JCO Precis Oncol* 2023;7:e2200715. 10.1200/PO.22.00715 37285561 PMC10309568

[12] Zalis M, Viana Veloso GG, Aguiar PN Jr et al. Next-generation sequencing impact on cancer care: applications, challenges, and future directions. *Front Genet* 2024;15:1420190. 10.3389/fgene.2024.1420190 39045325 PMC11263191

[13] Korkola J, Gray JW. Breast cancer genomes — form and function. *Curr Opin Genet Dev* 2010;20:4–14. 10.1016/j.gde.2009.11.005 20060285 PMC2956580

[14] Miki Y, Swensen J, Shattuck-Eidens D et al. A strong candidate for the breast and ovarian cancer susceptibility gene BRCA1. *Science* 1994;266:66–71. 10.1126/science.7545954 7545954

[15] Rouzier R, Perou CM, Symmans WF et al. Breast cancer molecular subtypes respond differently to preoperative chemotherapy. *Clin Cancer Res* 2005;11:5678–85. 10.1158/1078-0432.CCR-04-2421 16115903

[16] Hoadley KA, Yau C, Wolf DM et al. Multiplatform analysis of 12 cancer types reveals molecular classification within and across tissues of origin. *Cell* 2014;158:929–44. 10.1016/j.cell.2014.06.049 25109877 PMC4152462

[17] Fountzilas E, Pearce T, Baysal MA et al. Convergence of evolving artificial intelligence and machine learning techniques in precision oncology. *NPJ Digit Med* 2025;8:75. 10.1038/s41746-025-01471-y 39890986 PMC11785769

[18] Libbrecht MW, Noble WS. Machine learning applications in genetics and genomics. *Nat Rev Genet* 2015;16:321–32. 10.1038/nrg3920 25948244 PMC5204302

[19] Del Giudice M, Peirone S, Perrone S et al. Artificial intelligence in bulk and single-cell RNA-sequencing data to Foster precision oncology. *Int J Mol Sci* 2021;22:4563. 10.3390/ijms22094563 PMC8123853 33925407

[20] Bhinder B, Gilvary C, Madhukar NS et al. Artificial intelligence in cancer research and precision medicine. *Cancer Discov* 2021;11:900–15. 10.1158/2159-8290.CD-21-0090 33811123 PMC8034385

[21] Azuaje F . Artificial intelligence for precision oncology: beyond patient stratification. *NPJ Precis Oncol* 2019;3:6. 10.1038/s41698-019-0078-1 30820462 PMC6389974

[22] Huang S, Yang J, Shen N et al. Artificial intelligence in lung cancer diagnosis and prognosis: current application and future perspective. *Semin Cancer Biol* 2023;89:30–7. 10.1016/j.semcancer.2023.01.006 36682439

[23] Guo J, Hu J, Zheng Y et al. Artificial intelligence: opportunities and challenges in the clinical applications of triple-negative breast cancer. *Br J Cancer* 2023;128:2141–9. 10.1038/s41416-023-02215-z 36871044 PMC10241896

[24] Zeng Z, Mao C, Vo A et al. Deep learning for cancer type classification and driver gene identification. *BMC Bioinformatics* 2021;22:491. 10.1186/s12859-021-04400-4 34689757 PMC8543824

[25] Yan M, Gu Y, Sun H et al. Neutrophil extracellular traps in tumor progression and immunotherapy. *Front Immunol* 2023;14:1135086. 10.3389/fimmu.2023.1135086 36993957 PMC10040667

[26] Ma Y, Wei J, He W et al. Neutrophil extracellular traps in cancer. *MedComm (2020)* 2024;5:e647. 10.1002/mco2.647 39015554 PMC11247337

[27] Li W, Zhuang Y, Shao SJ et al. Essential contribution of the JAK/STAT pathway to carcinogenesis, lytic infection of herpesviruses and pathogenesis of COVID-19 (review). *Mol Med Rep* 2024;29:39. 10.3892/mmr.2024.13163 38240082 PMC10828999

[28] Baghaie L, Bunsick DA, Aucoin EB et al. Pro-inflammatory cytokines transactivate glycosylated cytokine receptors on cancer cells to induce epithelial-mesenchymal transition to the metastatic phenotype. *Cancers (Basel)* 2025;17:1234. 10.3390/cancers17071234 PMC11988151 40227834

[29] Tu T, McQuaid TJ, Jacobson IM. HBV-induced carcinogenesis: mechanisms, correlation with viral suppression, and implications for treatment. *Liver Int* 2025;45:e16202. 10.1111/liv.16202 39720865 PMC11669079

[30] Chávez-Domínguez RL, Torres M, Acevedo-Domínguez AA et al. Reprogramming the host: mycobacterium tuberculosis as a silent architect of the immuno-tumoral. *Front Cell Infect Microbiol* 2025;15:1697874. 10.3389/fcimb.2025.1697874 41322988 PMC12658415

[31] Rokade S, Damani AM, Oft M et al. IL-2 based cancer immunotherapies: an evolving paradigm. *Front Immunol* 2024;15:1433989. 10.3389/fimmu.2024.1433989 39114660 PMC11303236

[32] Im SJ, Lee K, Ha SJ. Harnessing IL-2 for immunotherapy against cancer and chronic infection: a historical perspective and emerging trends. *Exp Mol Med* 2024;56:1900–8. 10.1038/s12276-024-01301-3 39218982 PMC11447265

[33] Monteleone G, Pallone F, Stolfi C. The dual role of inflammation in colon carcinogenesis. *Int J Mol Sci* 2012;13:11071–84. 10.3390/ijms130911071 23109839 PMC3472731

[34] Rasch S, Algül H. A clinical perspective on the role of chronic inflammation in gastrointestinal cancer. *Clin Exp Gastroenterol* 2014;7:261–72. 10.2147/CEG.S43457 25143751 PMC4134025

[35] Liu C, Yin Q, Wu Z et al. Inflammation and immune escape in ovarian cancer: pathways and therapeutic opportunities. *J Inflamm Res* 2025;18:895–909. 10.2147/JIR.S503479 39867950 PMC11762012

[36] Martínez-Jiménez F, Muiños F, Sentís I et al. A compendium of mutational cancer driver genes. *Nat Rev Cancer* 2020;20:555–72. 10.1038/s41568-020-0290-x 32778778

[37] Furlan D, Carnevali IW, Bernasconi B et al. Hierarchical clustering analysis of pathologic and molecular data identifies prognostically and biologically distinct groups of colorectal carcinomas. *Mod Pathol* 2011;24:126–37. 10.1038/modpathol.2010.179 20852594

[38] Zhang Y, Du P, Li Y et al. TASP1 promotes gallbladder cancer cell proliferation and metastasis by up-regulating FAM49B via PI3K/AKT pathway. *Int J Biol Sci* 2020;16:739–51. 10.7150/ijbs.40516 32071545 PMC7019140

[39] Alim LF, Keane C, Souza-Fonseca-Guimaraes F. Molecular mechanisms of tumour necrosis factor signalling via TNF receptor 1 and TNF receptor 2 in the tumour microenvironment. *Curr Opin Immunol* 2024;86:102409. 10.1016/j.coi.2023.102409 38154421

[40] Wu Y, Zhou BP. TNF-alpha/NF-kappaB/snail pathway in cancer cell migration and invasion. *Br J Cancer* 2010;102:639–44. 10.1038/sj.bjc.6605530 20087353 PMC2837572

[41] Ma K, Wang Y, Zhang Y et al. Clinical practice of targeted capture sequencing to identify actionable alterations in cholangiocarcinoma. *Cancers (Basel)* 2022;14:5062. 10.3390/cancers14205062 PMC9600135 36291846

[42] Li X, Tang Z, Li Z et al. Somatic mutations that affect early genetic progression and immune microenvironment in gastric carcinoma. *Pathol Res Pract* 2024;257:155310. 10.1016/j.prp.2024.155310 38663178

[43] Song M, Cheng H, Zou H et al. Genomic profiling informs therapies and prognosis for patients with hepatocellular carcinoma in clinical practice. *BMC Cancer* 2024;24:673. 10.1186/s12885-024-12407-2 38825709 PMC11145829

[44] Su H, Wang Y, Chao X et al. The impact of homologous recombination deficiency on the prognosis of epithelial ovarian cancer. *Clin Transl Med* 2025;15:e70143. 10.1002/ctm2.70143 39724505 PMC11670307

